# Histocompatibility Minor 13 (HM13), targeted by miR-760, exerts oncogenic role in breast cancer by suppressing autophagy and activating PI3K-AKT-mTOR pathway

**DOI:** 10.1038/s41419-022-05154-4

**Published:** 2022-09-25

**Authors:** Haiyan Yang, Zhi Li, Zhangwei Wang, Xu Zhang, Xinyuan Dai, Guoren Zhou, Qiang Ding

**Affiliations:** 1 Jiangsu Breast Disease Center, the First Affiliated Hospital with Nanjing Medical University, 300 Guangzhou Road, Nanjing, Jiangsu 210029 PR China; 2 Department of Breast and Thyroid Surgery, Huai’an First People’s Hospital, Nanjing Medical University, Huai’an, Jiangsu 223399 PR China; 3 Department of Oncology, Jiangsu Cancer Hospital & the Affiliated Cancer Hospital of Nanjing Medical University & Jiangsu Institute of Cancer Research, Nanjing, Jiangsu 210009 PR China

**Keywords:** Breast cancer, RNAi, Oncogenes, Genetics research, Cancer models

## Abstract

Histocompatibility Minor 13 (HM13) is reported to participate in regulating multiple cancers. In the present study, we uncovered that HM13 was highly expressed in breast cancer and correlated with worse prognosis. Downregulation of HM13 could suppress breast cancer cell proliferation and metastasis abilities. Tumorigenicity mediated by HM13 was also observed in the xenograft model. Knockdown of HM13 could activate autophagy by inducing endoplasmic reticulum (ER) stress. Moreover, further experiments demonstrated that downregulated HM13 could inhibit PI3K-AKT-mTOR pathway. We then verified that HM13 was a direct target of miR-760 functioned as a tumor -suppressor in breast cancer. And the tumor suppressive effects of miR-760 could be partially reversed by HM13. Taken together, these findings elucidated that HM13, targeted by miR-760, could play an oncogenic role in breast cancer by inducing autophagic inhibition and facilitating PI3K-AKT-mTOR pathway. Our findings suggested HM13 could act as a novel therapeutic target candidate for breast cancer and supported the idea that autophagy inducers might represent a new approach to treat breast cancer.

## Introduction

Breast cancer is the most common malignancy in females worldwide [1–4] in spite of the outstanding advances in the diagnosis and treatment in recent decades [1, 5]. Hence, broad public were motivated to explore the underlying mechanisms and discover new diagnostic markers for breast cancer.

HM13, a GXGD-type aspartyl I-CLIP localized to endoplasmic reticulum (ER), is also called as signal peptide peptidase (SPP) [6, 7]. In pathological condition, unfolded protein accumulates in the ER, which is named ER stress [8–10]. Upregulation of unfolded protein is often observed in tumors, indicating the occurrence of ER stress in cancer development [11, 12]. Based on TCGA database, we found that the expression of HM13 was significantly aberrant in breast cancer tissues. However, whether the dysregulated expression of HM13 is relevant to ER-stress remains unknown.

Autophagy is critical for maintaining the cellular homeostasis [13]. In different malignancies, it might be activated to alleviate ER stress and clear the accumulated misfolded proteins from the ER lumen [14]. For example, inhibition of BRAF (v-raf murine sarcoma viral oncogene homolog B) could disturb the normal protein folding machinery in the ER, leading to ER stress-mediated autophagy [15]. Autophagy could play neutral, tumor-suppressive, or tumor-promoting roles depending on type, stage or genetic context of the cancers [16, 17]. Recent reports showed that autophagy might be involved in hepatic cell carcinoma (HCC) metastasis through facilitating anoikis resistance and lung colonization of HCC cells [18], while inhibition of PHLDA2 (pleckstrin homology-like domain family A member 2) could increase apoptosis in colorectal cancer cells partly through the activation of autophagy [19]. However, the underlying mechanism by which autophagy mediates breast cancer progression remains to be elucidated. Several studies showed that complex signal transduction pathways might be in the involvement of autophagy regulation, among which PI3K-AKT-mTOR pathway was considered as a negative regulation of autophagy [20, 21]. PI3K-AKT-mTOR pathway maintained close contacts with cell proliferation, growth, metabolism, and motility [22]. Previous studies illustrated that inhibition of SOCS5 (Suppressor of cytokine signaling 5) could suppress HCC cell migration and invasion in vitro by activating PI3K-Akt-mTOR-mediated autophagy [23], while overexpressing IMPDH2 (Inosine 5’-monophosphate dehydrogenase 2) could facilitate cell invasion, migration and epithelial mesenchymal transformation (EMT) by regulating PI3K-AKT-mTOR pathway in colorectal cancer [24]. Yet, whether HM13 promotes breast cancer progression via PI3K-AKT-mTOR signaling has been rarely investigated.

One of the most common post-transcriptional regulation of gene mechanism is the regulation by related microRNAs (miRNAs) [25, 26]. miRNAs are a class of small non-coding RNAs with a length of ~22 nucleotides [27]. They could downregulate the targeted mRNAs expression by binding to 3′-untranslated regions (3′-UTR), indicating that miRNAs may act as both tumor suppressors and oncogenic factors [28, 29]. For instance, miR-195-5p regulated Notch2-mediated tumor cell EMT by directly binding to the 3’-UTR of the Notch2 mRNA in colorectal cancer [30]. Moreover, miR-324-3p mediated gastric cancer progression through activating the Smad4-mediated Wnt/beta-catenin signaling pathway [31]. Accordingly, bioinformatics and functional assays indicated that HM13 might be regulated by miR-760.

In the present study, HM13 was identified to have an oncogenic function in breast cancer through autophagy triggered by ER-stress. PI3K-AKT-mTOR pathway was involved in breast cancer progression. Mechanistically, miR-760 negatively regulated HM13 mRNA expression and made suppressive effects on breast cancer. The findings suggested HM13 could act as a novel therapeutic target candidate for breast cancer and supported the idea that autophagy inducers might represent a new approach to breast cancer.

## Results

### HM13 is upregulated in human breast cancer and correlated with poorer breast cancer prognosis

**Fig. 1:**
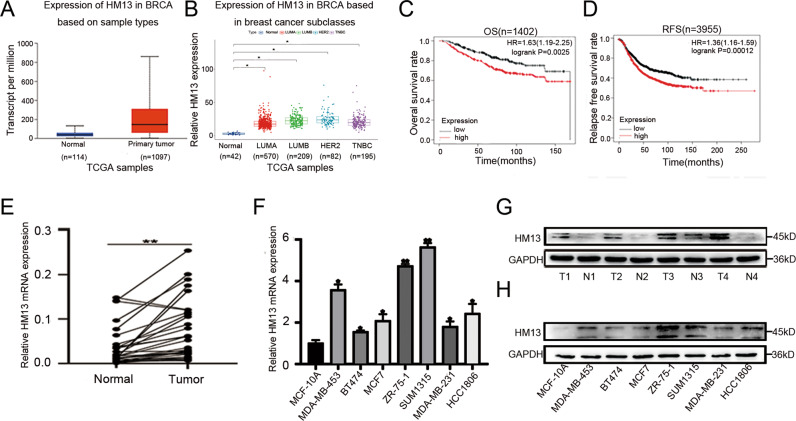
HM13 is up-regulated in breast cancer. **A**, **B** The expression of HM13 according to TCGA based on sample types (**A**) or subclasses (**B**) in breast cancer. Transcriptome profiling was obtained from TCGA. Based on PAM50 signature, we processed the data and showed the expression level of HM13 in breast cancer subclasses. *P*-values smaller than 0.05. **C**, **D** The correlation between HM13 expression and prognosis of the breast cancer patients was presented by Kaplan-Meier survival analysis. **E**, **F** qRT-PCR was used to evaluate the mRNA expression levels of HM13 in breast cancer tissues (**E**) and cell lines (**F**), respectively. **G**, **H** Protein expression of HM13 in pairs of tissues (**G**) and cell lines (**H**) of breast cancer. Data were shown as mean ± SD, *p < 0.05, **p < 0.01.

As shown in Fig. 1A, B, HM13 displayed a high level of expression based on sample types, as well as each subtype of breast cancer. Kaplan Meier plot analysis suggested that higher HM13 expression level was correlated with worse prognosis of breast cancer patients (Figs. 1C, D, S1A, B). As illustrated in Table 1, highly expressed HM13 in breast cancer was related to a late T grade and a poor histological type of breast cancer. The mRNA expression (Fig. 1E, F) and protein level (Fig. 1G, H, Fig. S7A, B) of HM13 were higher in the tumor tissues and breast cancer cell lines (especially in SUM1315 and ZR-75-1) than in the adjacent normal tissues and normal mammary epithelial cell line (MCF-10A).

**Table 1:** Expression of HM13 in human breast cancer patients’ tissues according to patients’ clinicopathological.

Characteristic	Low expression of HM13	High expression of HM13	*P*-value
*n*	541	542	
Age, *n* (%)			0.973
<=60	301 (27.8%)	300 (27.7%)	
>60	240 (22.2%)	242 (22.3%)	
T stage, *n* (%)			**0.001**^**※**^
T1	156 (14.4%)	121 (11.2%)	
T2	298 (27.6%)	331 (30.6%)	
T3	77 (7.1%)	62 (5.7%)	
T4	9 (0.8%)	26 (2.4%)	
N stage, *n* (%)			0.356
N0	261 (24.5%)	253 (23.8%)	
N1	182 (17.1%)	176 (16.5%)	
N2	50 (4.7%)	66 (6.2%)	
N3	42 (3.9%)	34 (3.2%)	
M stage, *n* (%)			0.455
M0	460 (49.9%)	442 (47.9%)	
M1	8 (0.9%)	12 (1.3%)	
Pathologic stage, *n* (%)			0.237
Stage I	102 (9.6%)	79 (7.5%)	
Stage II	302 (28.5%)	317 (29.9%)	
Stage III	119 (11.2%)	123 (11.6%)	
Stage IV	7 (0.7%)	11 (1%)	
Histological type, *n* (%)			**<** **0.001**^**※**^
Infiltrating Ductal Carcinoma	347 (35.5%)	425 (43.5%)	
Infiltrating Lobular Carcinoma	147 (15%)	58 (5.9%)	

The bold values meant that *P* value was smaller than 0.05 and it was considered significant. To be specific, highly expressed HM13 in breast cancer was related to a later T grade and a poorer histological type of breast cancer.

*P* ≤ 0.05 was considered significant. ^※^*P* values smaller than 0.05.

### HM13 promotes breast cancer proliferation and metastasis

**Fig. 2:**
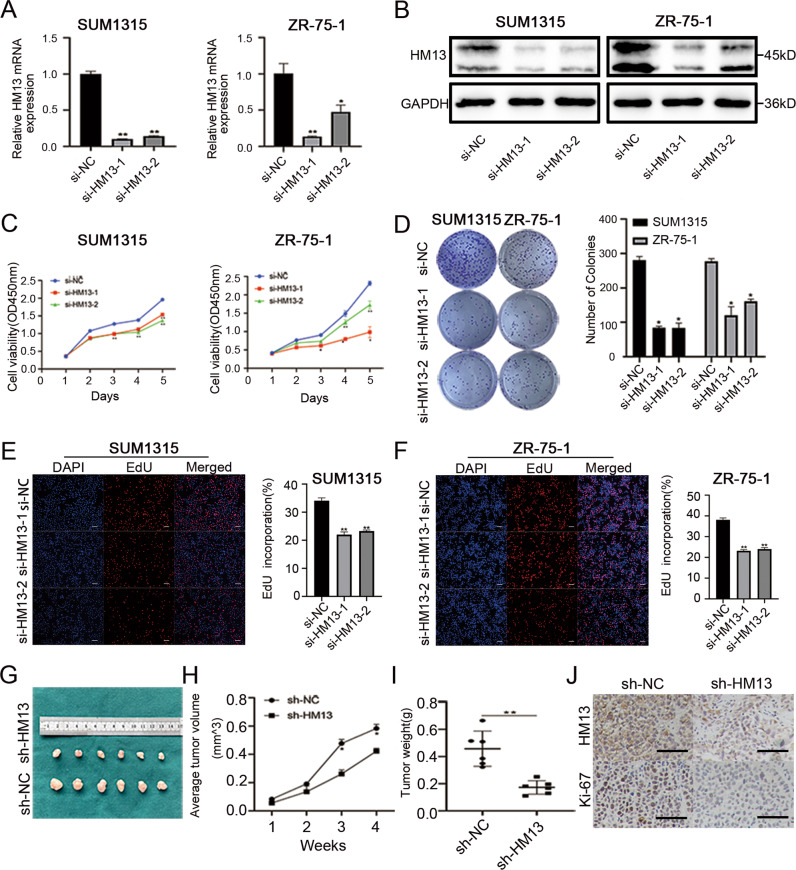
Downregulation of HM13 suppresses the proliferation of breast cancer in vitro and in vivo. **A**, **B** The transfection efficiencies of si-HM13-1 and si-HM13-2 were evaluated by qRT-PCR (**A**) and western blot (**B**) in SUM1315 and ZR-75-1 cell lines. **C** The CCK-8 assays were performed to measure the cell viability of SUM1315 (left) and ZR-75-1 (right) cell lines transfected with siRNAs. **D** Representative results of the colony formation showed the cell proliferation in SUM1315 and ZR-75-1 cell lines after downregulation of HM13. **E**, **F** EdU assays were conducted to compare the growth rates in SUM1315 (**E**) and ZR-75-1 (**F**) cell lines between experimental groups (si-HM13-1 and si-HM13-2) and control group (si-NC). DAPI was indicated by blue, EdU was indicated by red. Scale bars, 50 μm. **G** Images of xenograft tumors from nude mice classified into HM13 knockdown (sh-HM13) group and control (sh-NC) group (*n* = 6). **H**, **I** Average tumor volume (**H**) and tumor weight (**I**) of breast cancer in knockdown of HM13 (sh-HM13) and control (sh-NC) group were shown by tumor growth curves. **J** IHC staining of breast cancer tissues from tumor subcutaneous mice model was aimed to determine the positive rates of HM13 and Ki-67 in knockdown of HM13 (sh-HM13) and control (sh-NC) group. Scale bars, 100 µm. Data were shown as mean ± SD, (*p < 0.05, **p < 0.01, ***p < 0.001).

To explore the biological functions of HM13 in breast cancer, two siRNAs were transfected into SUM1315 and ZR-75-1 cell lines. The results showed that HM13 expression was inhibited at the mRNA expression (Fig. 2A) and the protein level (Fig. 2B, Fig. S7C). According to the growth curves derived from the CCK-8 assay (Fig. 2C), knockdown of HM13 could slow down the growth rate of breast cancer cells, in line with the results of the colony formation (Fig. 2D) and EdU assays (Fig. 2E, F). Moreover, to validate the effects of HM13 on breast cancer proliferation in vivo, the stably transfected SUM1315 cells were subcutaneously injected into nude mice. The results showed that downregulated HM13 could suppress the breast cancer proliferation in vivo (Fig. 2G–I). In addition, IHC staining derived from breast cancer tissues of tumor subcutaneous mice model demonstrated that the positive rates of HM13 and Ki-67 were reduced in the HM13 knockdown group, compared to that of the control group (Fig. 2J). Ki-67 protein has been widely used as a proliferation marker for human tumor cells [32]. An opposite effect (Fig. S2C–J) was found when HM13 was overexpressed (Figs. S2A, B, S7J).

The effects of HM13 on breast cancer cells metastasis were next investigated. Wound healing assays (Fig. S3A, B), as well as transwell assays (Fig. S3C, D) elucidated that downregulated HM13 could suppress the migration and invasion abilities of breast cancer, while HM13 overexpression could facilitate breast cancer metastasis (Fig. S3E–H). Taken together, HM13 could accelerate breast cancer progression.

### Downregulation of HM13 activates autophagy by inducing ER-stress

**Fig. 3:**
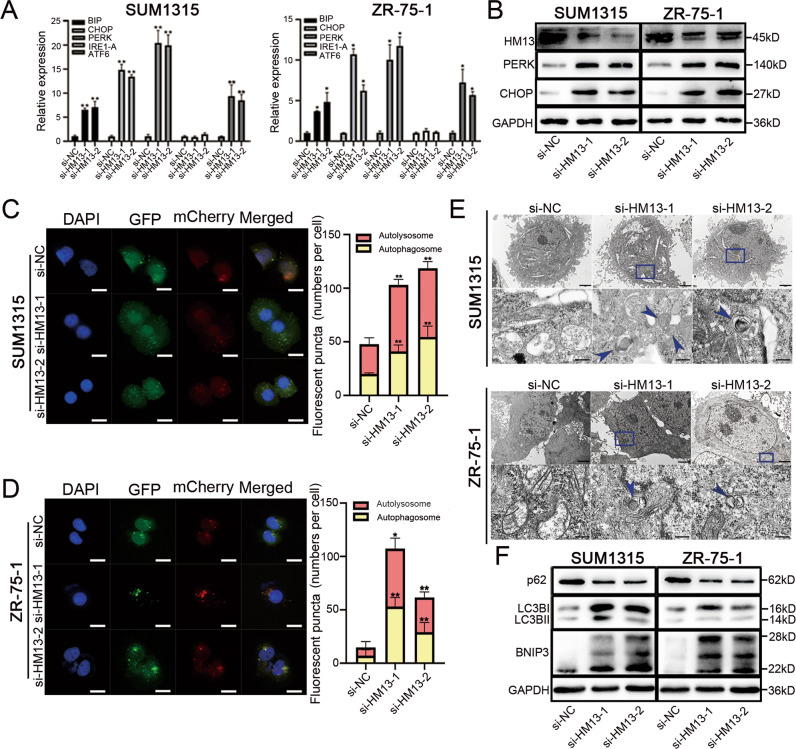
Knockdown of HM13 triggers autophagy by inducing ER-stress in breast cancer. **A** SUM1315 (left) and ZR-75-1 (right) cell lines were transfected with siRNAs (si-HM13-1 and si-HM13-2) or negative control (si-NC) and relative mRNA expression of ER stress-relating genes was analyzed by qRT–PCR. **B** Western blot was performed to detect the protein levels of PERK and CHOP in breast cancer cell lines in HM13 knockdown groups and control group. **C**, **D** SUM1315 (**C**) and ZR-75-1 (**D**) were stably transfected with mCherry-EGFP-LC3B lentivirus and then treated with siRNAs or si-NC, respectively. The subcellular localization of autophagosomes and autolysosomes were illustrated by fluorescent confocal microscopy and the bars graphs showed the specific quantities of the fluorescent puncta data. The yellow puncta indicated autophagosomes, and the red puncta indicated autolysosomes (the fusion of autophagosomes with lysosomes) in the merged figures. The data were presented as the mean ± SD of three experiments. Scale bars, 10 µm. **E** Representative transmission electron micrographs demonstrated the ultrastructure of breast cancer cells. Double membrane autophagosomes were counted in randomly selected >100 cells. Arrow indicates the autophagosomes distributing in the cytoplasm. Scale bars, 2 µm and 200 nm. **F** The protein expressions of LC3B-II, LC3B-I, and p62 in SUM1315 and ZR-75-1 respectively transfected with siRNAs or negative control were determined by western blot. Data were shown as mean ± SD, *p < 0.05, **p < 0.01, ***p < 0.001.

SPP, encoded by HM13, is an ER-resident presenilin-type aspartic protease [6]. In pathological conditions, the destruction of the physiological function of ER leads to the accumulation of unfolded protein in the lumen of the ER-namely, ER stress [8, 33]. ER stress has been established as an important mechanism of cell survival and tumor progression [9, 34, 35]. Therefore, we investigated the relationship between HM13 and ER-stress in breast cancer cell lines. As shown in Fig. 3A, the mRNA expression of pathway genes related to ER-stress, protein kinase RNA-like endoplasmic reticulum kinase (PERK) and activating transcription factor 6 (ATF6) significantly increased in each experimental group, as well as the marker genes immunoglobulin heavy chain binding protein in pre-B cells (BIP) and transcription factor C/EBP-homologous protein (CHOP). Meanwhile, the protein levels of PERK and CHOP in SUM1315 and ZR-75-1 cell lines also increased in HM13 downregulated groups (Fig. 3B, Fig. S7D). Moreover, autophagy activated by ER stress is a key regulatory mechanism to regulate the biological function of cells [33, 36]. We next utilized the mCherry-EGFP-LC3B reporter to monitor autophagosomes and autolysosomes whose GFP signal is vulnerable to acidic conditions after autolysosome formation, whereas the mCherry signal is less affected. Therefore, the yellow puncta indicate autophagosomes, and the red puncta indicate autolysosomes (the fusion of autophagosomes with lysosomes) in the merged figures. The formation of both autophagosomes and autolysosomes increased in breast cancer cells transfected with siRNAs (si-HM13-1 and si-HM13-2) (Fig. 3C, D). It indicated the knockdown of HM13 could trigger autophagic synthesis in breast cancer cells. Autophagosomes, characterized with double-membraned autophagic vacuoles indicated the activation of autophagy. The results of TEM (Fig. 3E) indicated that the numbers of autophagosomes increased in SUM1315 and ZR-75-1 cell lines, when HM13 expression was reduced. Furthermore, downregulated HM13 in the same set of cell lines could increase the protein levels of LC3B-II/I together with BNIP3 and decrease the protein level of p62 (Fig. 3F, Fig. S7E). To sum up, downregulation of HM13 could facilitate the autophagy via activating ER-stress.

### HM13 regulates the proliferation and metastasis in breast cancer through autophagy

**Fig. 4:**
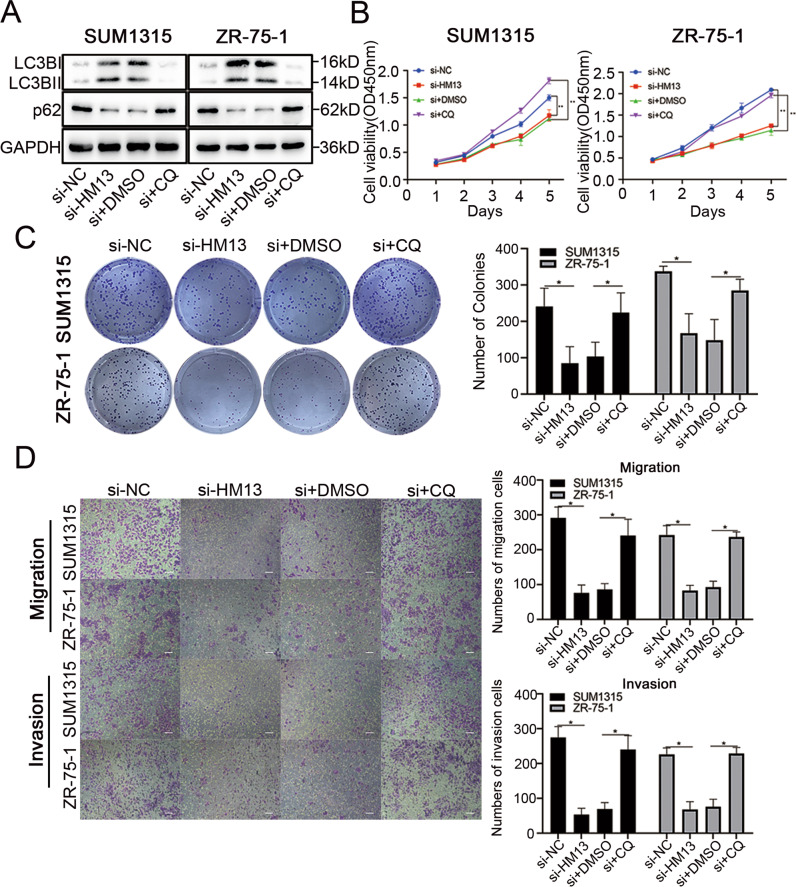
HM13 regulates the biological functions in breast cancer through autophagy. **A** In SUM1315 and ZR-75-1 cell lines transfected with negative control (si-NC), siRNA (si-HM13), siRNA and DMSO (si+DMSO), siRNA and CQ (si+CQ), the protein levels of LC3B-II/I and p62 were analyzed by western blot, respectively. **B**, **C** Cell proliferation abilities were determined by CCK8 (**B**) and colony formation assays (**C**). **D** Representative images of the transwell assays using SUM1315 an ZR-75-1 cell lines transfected with negative control (si-NC), siRNA (si-HM13), siRNA and DMSO (si + DMSO), siRNA and CQ (si + CQ). Scale bars, 50 µm. Data were shown as mean ± SD, *p < 0.05, **p < 0.01.

To detect whether HM13 promotes breast cancer progression through autophagy, we conducted a series of functional experiments. Breast cancer cells were treated with 25 μM chloroquine (CQ), an autophagolysosome fusion inhibitor. The co-treatment with si-HM13 and CQ could decrease LC3B-II/I ratio and increase p62 level compared to the corresponding group (Fig. 4A, Fig. S7F). CCK-8 assays (Fig. 4B), together with colony formation (Fig. 4C) assays elucidated that the treatment of CQ could reverse the inhibition of cell proliferation induced by downregulated HM13 in SUM1315 and ZR-75-1 cell lines. Moreover, according to the transwell assays, the numbers of metastasis cells increased in the group with the addition of CQ (Fig. 4D). These results proved that HM13 could promote breast cancer proliferation and metastasis in vitro through autophagy.

### HM13 accelerates breast cancer progression via activating PI3K-AKT-mTOR pathway

**Fig. 5:**
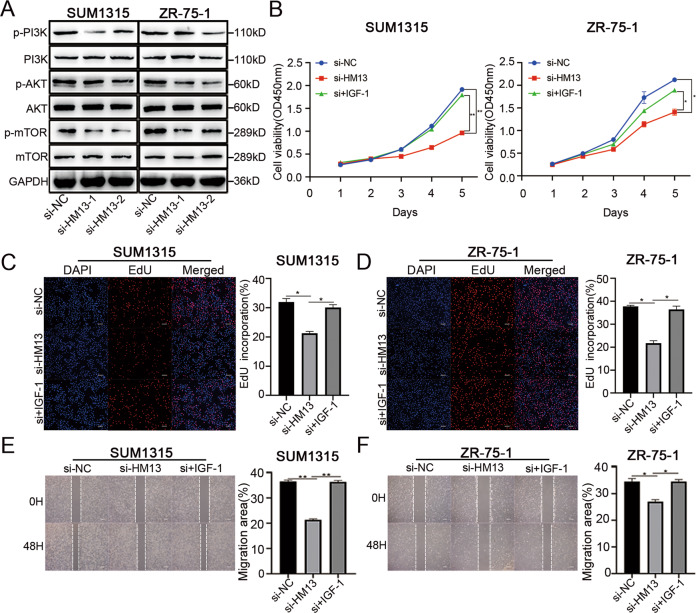
Downregulated HM13 inhibits PI3K-AKT-mTOR pathway in breast cancer. **A** Western blot assessment of PI3K, p-PI3K, AKT, p-AKT, mTOR, p-mTOR protein expression was performed in SUM1315 and ZR-75-1 cell lines with siRNAs (si-HM13-1 and si-HM13-2) or negative control (si-NC). **B**, **D** The effects of IGF-1 on cell proliferation were detected by CCK-8 (**B**) and EdU (**C**, **D**) assays. SUM1315 and ZR-75-1 cell lines were transfected with negative control (si-NC), siRNA (si-HM13) or siRNA and IGF-1 (si+IGF-1) before harvesting. Scale bars, 50 µm. **E**, **F** The wound healing assays were performed to determine the migration abilities of SUM1315 (**E**) and ZR-75-1 (**F**) transfected with negative control (si-NC), siRNA (si-HM13) or siRNA and IGF-1 (si+IGF-1). Scale bars, 100 µm. Data were shown as mean ± SD, *p < 0.05, **p < 0.01.

The previous study indicated that FKBP8, functioned as an endogenous mTOR inhibitor, was degraded by SPP-mediated intramembrane cleavage [37]. Our western blot results also showed that HM13 could facilitate FKBP8 protein degradation (Figs. S6, S7K). PI3K-AKT-mTOR pathway, which plays a central role in keeping cellular and physiological homeostasis in cancers, has been considered as a negative regulation of autophagy [38, 39]. Insulin-like growth factor I (IGF-1), as a stimulator for AKT, was conducted to confirm the involvement of PI3K-AKT-mTOR pathway in the tumorigenicity of HM13. The phosphorylated levels of PI3K-AKT-mTOR pathway-related marker genes decreased in breast cancer cell lines, when HM13 expression was reduced (Fig. 5A, Fig. S7G). CCK-8 assay (Fig. 5B), together with EdU assay (Fig. 5C, D) suggested that IGF-1 reversed the inhibition of breast cancer cell proliferation caused by the knockdown of HM13. Meanwhile, the wound healing assays indicated that IGF-1 could partly promote the migration area inhibited by the downregulated HM13 (Fig. 5E, F). These results uncovered that HM13 could accelerate breast cancer progression via activating PI3K-AKT-mTOR pathway.

### miR-760 negatively targets and regulates HM13 in breast cancer

**Fig. 6:**
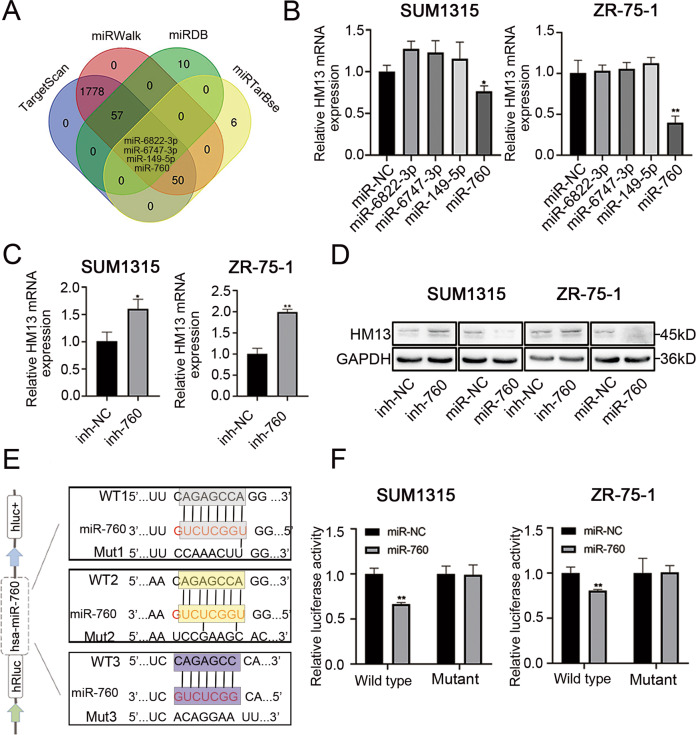
miR-760, as a negative factor, targets HM13 in breast cancer. **A** The potential candidate miRNAs targeting HM13 were screened and presented by the Venn-diagram. **B** The relative mRNA expression of HM13 was analyzed by qRT-PCR. miR-6822-3p mimics (miR-6822-3p), miR-6747-3p mimics (miR-6747-3p), miR-149-5p mimics (miR-149-5p) or miR-760 mimics (miR-760) was transfected into breast cancer cell lines for 48 h before harvesting, respectively. **C** The mRNA expression of HM13 in SUM1315 (left) and ZR-75-1 (right) cell lines after transfected with miR-760 inhibitor (inh-760) and inhibitor control (inh-NC) was determined by qRT-PCR. **D** Relative protein expression of HM13 from four groups containing miR-760 inhibitor (inh-760) group, miR-760 mimics (miR-760) group and their corresponding control groups (inh-NC and miR-NC) was evaluated through western blot. **E** Schematic diagram of the regions in the 3′-UTR of HM13 mRNA. **F** Relative luciferase activities of wild type and mutant HM13 reporter plasmid in SUM1315 (left) and ZR-75-1 (right) cell lines transfected with miR-760 mimics (miR-760) or mimics control (miR-NC). Data were shown as mean ± SD, *p < 0.05, **p < 0.01.

The underlying mechanism between miRNA dysregulation and the rationale of aberrant HM13 expression in breast cancer was further investigated. Four algorithms (TargetScan, mirWalk, mirRDB, and miTarBase) were used to screen and predict the putative miRNAs targeting HM13 gene in breast cancer. miR-6822-3p, miR-6747-3p, miR-149-5p and miR-760 were identified as potential candidates via bioinformatics analysis (Fig. 6A). We then upregulated the candidate miRNAs and found only miR-760 could reduce the mRNA expression of HM13 in SUM1315 and ZR-75-1 cell lines (Fig. 6B). More than that, the mRNA expression level of HM13 was found to be upregulated in the same set of cell lines transfected with miR-760 inhibitor (Fig. 6C). The similar results were also verified through western blot in breast cancer cells (Fig. 6D, Fig. S7H). As illustrated in Fig. 6E, F, the relative luciferase activity of wild type, not mutant vector, was obviously reduced after co-transfection of miR-760 mimics in SUM1315 and ZR-75-1 cell lines, implying that HM13 was a direct target of miR-760 in breast cancer cells.

### miR-760, a tumor-suppressing role in breast cancer

Given that miR-760 directly aimed at HM13, a series of assays were conducted to examine its molecular function and biological process in breast cancer. miR-760 expression in breast cancer tissues was significantly lower than that in their adjacent normal tissues (Fig. S4A). Moreover, miR-760 expression was inversely correlated with HM13 expression in 30 pairs of breast cancer tissues (Fig. S4B). Upregulated miR-760 could decrease cell viability in breast cancer cells, while miR-760 inhibition could increase cell viability (Fig. S4C, D), which was verified again via EdU assay (Fig. S4E, F). Similarly, the same cell lines treated with miR-760 mimics weakened the abilities of wound healing in breast cancer cells, whereas miR-760 inhibitor reversed the effects (Fig. S5A, B). In addition, the results from transwell assays showed a decrease in breast cancer cells migration and invasion abilities when miR-760 expression was upregulated. However, the metastasis abilities of SUM1315 and ZR-75-1 cell lines were enhanced by transfecting miR-760 inhibitor (Fig. S5C, D). These data illustrated that miR-760 functioned as a tumor-suppressing role in the regulation of breast cancer progression.

### miR-760 mediates autophagy and PI3K-AKT- mTOR pathway by targeting HM13

**Fig. 7:**
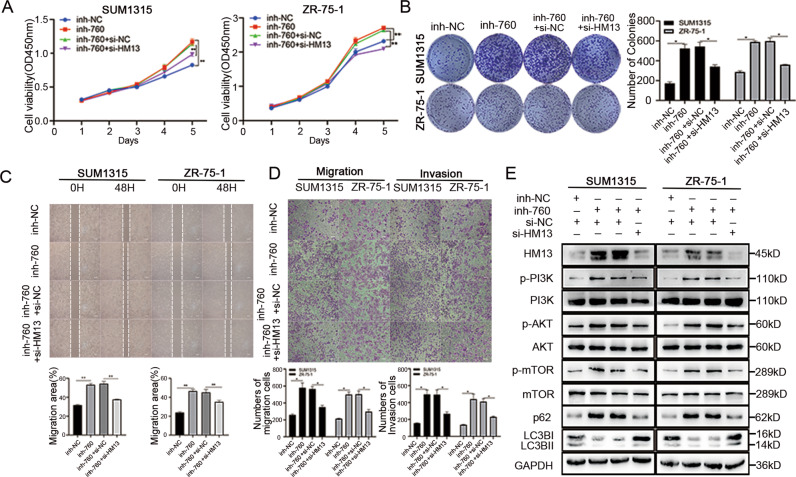
miR-760 is involved in autophagy and PI3K-AKT-mTOR signaling via targeting HM13. **A**, **B** Inhibitor control (inh-NC), miR-760 inhibitor (inh-760), miR-760 inhibitor and negative control (inh-760+si-NC) or miR-760 inhibitor and siRNA (inh-760+si-HM13) was transfected into SUM1315 and ZR-75-1 cell lines, respectively. The cell proliferation abilities were evaluated via CCK-8 (**A**), as well as colony formation assay (**B**). **C**, **D** SUM1315 and ZR-75-1 cell lines were divided into four groups, inhibitor control (inh-NC) group, miR-760 inhibitor (inh-760) group, miR-760 inhibitor and negative control (inh-760+si-NC) group or miR-760 inhibitor and siRNA (inh-760+si-HM13) group. The metastasis abilities were detected by the wound healing assays (**C**) and the transwell assays (**D**). **E** Western blot was aimed to evaluate the protein expression of genes related to autophagy and PI3K-AKT-mTOR signaling in rescue experiments. Data were shown as mean ± SD, *p < 0.01, **p < 0.01.

We performed rescue experiments to detect whether miR-760 functionally targeted HM13 in breast cancer. The CCK-8 (Fig. 7A), as well as the colony formation assays (Fig. 7B) suggested the promotive effects caused by miR-760 inhibitor in proliferation ability could be partly reversed when HM13 expression was reduced. The wound healing assays manifested the healing speed of scratches was faster in breast cancer cells transfected with miR-760 inhibitor and si-HM13 (Fig. 7C). Similarly, the results derived from the transwell assays (Fig. 7D) were in accordance with the wound healing assays, indicating the increase of metastasis abilities caused by miR-760 inhibitor in SUM1315 and ZR-5-1 cell lines could be partly restored when HM13 expression was reduced. These findings demonstrated the role of HM13 in promoting breast cancer proliferation and metastasis is primarily dependent on the miR-760/HM13 axis.

**Fig. 8:**
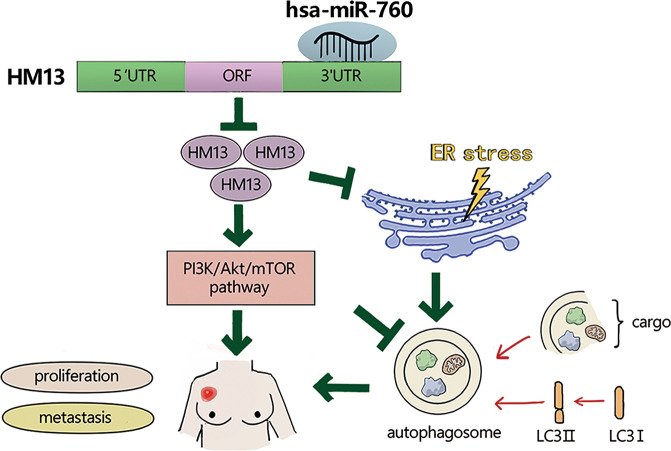
A schematic diagram showing HM13, targeted by miR-760, exerts oncogenic role in breast cancer through autophagy and PI3K-AKT-mTOR signaling. Downregulated HM13 could activate autophagy by inducing ER-stress, while HM13 upregulation could facilitate PI3K-AKT-mTOR pathway in breast cancer. Moreover, miR-760 negatively targets HM13 by binding to 3′-UTR of its mRNA. Thus, HM13 plays an oncogenic role in breast cancer progression.

Our previous results revealed that downregulation of HM13 could activate autophagy and inhibit PI3K-AKT- mTOR pathway. To investigate whether miR-760 could regulate these effects by targeting HM13, we assessed the protein levels of genes relevant to autophagy and PI3K-AKT-mTOR signaling in SUM1315 and ZR-75-1 cell lines. It illustrated that miR-760 inhibitor could decrease LC3B-II/I and increase the protein level of p62. Nevertheless, these changes could be restored by HM13 knockdown (Fig. 7E, Fig. S7I). Meanwhile, increase of the levels of the phosphorylation of mTOR, PI3K and AKT induced by miR-760 inhibitor was also partly recovered by the knockdown of HM13 (Fig. 7E, Fig. S7I). Therefore, miR-760 could mediate autophagy and PI3K-AKT- mTOR pathway by targeting HM13 in breast cancer (Fig. 8).

## Discussion

In the present study, we performed a series of experiments to elucidate the function and underlying molecular mechanism of HM13 in breast cancer. After the analysis in TCGA together with the validation in cell lines and tumor tissues, we found the abnormally high expression level of HM13 in breast cancer. Functional investigations indicated that HM13 exerted its oncogenic role by promoting the proliferation and metastasis of breast cancer cells. In vivo, a high expression level of HM13 was correlated with a large tumor size and a high tumor weight. These results indicated that HM13 might be a potential biomarker and therapeutic target for breast cancer patients.

Downregulated HM13 activated autophagy induced by ER-stress. Previous studies have been documented that ER-stress is correlated with progression of different types of cancer [40]. Autophagy could be effectively induced by ER-stress [33, 41]. Under the circumstance of autophagy, the malignant tumor cells maintain growth by re-using their organelles [42]. In our study, ER-stress related genes, such as PERK and CHOP significantly increased when HM13 expression was reduced. Downregulated HM13 could promote autophagy, demonstrated by following results, the formation of autophagosomes viewed through fluorescent confocal microscopy and transmission electron microscopy, upregulation of the autophagy marker protein LC3B-II/I, and downregulation of p62. It was worth noting that the number of red puncta increased more than that of yellow puncta, indicating autolysosomes increased more than autophagosomes. This might be related to the underlying mechanism between HM13 and autophagy flow, which remains to be further explored in future studies. Although the autonomous functions of autophagy in tumor cells have been demonstrated to promote survival and metabolic adaptation by recycling essential metabolites and amino acids, it is now well-recognized that autophagy can play neutral, tumor-suppressive, or tumor-promoting roles depending on type, stage or genetic context of the cancers [43]. The inhibition of cell proliferation and metastasis abilities in HM13 downregulated breast cancer cells could be reversed by CQ. Altogether, these results provided evidence that HM13 exerted its oncogenic role by targeting autophagy. PI3K-AKT-mTOR pathway, as a critical regulator of autophagy [44, 45], is deregulated in various human diseases, especially in cancers [46, 47]. Our studies demonstrated that knockdown of HM13 inhibited PI3K-AKT-mTOR pathway. IGF-1, a stimulator for AKT, could reverse the inhibitory effects caused by knockdown of HM13. Our observation revealed that HM13 could accelerate breast cancer progression via activating PI3K-AKT-mTOR pathway. The previous study indicated that FKBP8, functioned as an endogenous mTOR inhibitor [48], was degraded by SPP-mediated intramembrane cleavage [37]. Our further experiment demonstrated that HM13 could facilitate FKBP8 protein degradation. mTOR could activate PI3K/Akt signaling [21]. Meanwhile, our western blot analysis showed that downregulation of HM13 could inhibit mTOR. Therefore, we suspected that HM13 might upregulate mTOR by facilitating FKBP8 degradation, which could promote PI3K/Akt signaling.

The abnormally high expression of HM13 motivated us to explore why it was upregulated in breast cancer. Gene expression could be modulated by miRNAs by the way of promoting mRNA degradation or inhibiting mRNA translation at the post-transcriptional level [49]. HM13 was identified as a potential target of miR-760, which was confirmed by bioinformatics analysis and luciferase reporter assay. miRNA dysregulation correlates with various cancers by acting as tumor suppressors and oncogenes [50]. Previous studies demonstrated that miR-760 may act as a tumor suppressor gene in non-small cell lung cancer (NSCLC), colorectal cancer and gastric cancer [51–53]. Our data illustrated that miR-760 functioned as an endogenous inhibitor in breast cancer. The mRNA expression of 30 pairs of breast cancer tissue samples was detected and we found the negative correlations between HM13 and miR-760. Moreover, miR-760 exerted its suppressive role by targeting HM13. Downregulated HM13 recovered miR-760 inhibitor-inducing autophagy suppression and PI3K-AKT-mTOR pathway activation. This new miR-760/HM13 axis enhanced our understanding of breast cancer progression.

In summary, our work confirmed that HM13 was highly expressed in breast cancer, at least in part, by facilitating the degradation of autophagosomes. HM13 also exerted an oncogenic role by triggering the PI3K-AKT-mTOR signaling. Moreover, our characterization of this new miR-760/HM13 axis enhanced our understanding of breast cancer progression. Obviously, the accumulated evidence highlights the potential of HM13 as a novel therapeutic target for breast cancer treatment. Moreover, it becomes evident that autophagy inducers could be translated into clinical approach to the treatment of breast cancer.

## Materials and methods

### Breast cancer tissue samples

Breast cancer tissues and adjacent normal tissues were obtained from the First Affiliated Hospital of Nanjing Medical University. All patients who were diagnosed with breast cancer based on histopathological evaluation received no neoadjuvant therapy. The Institutional Ethics Committee of the First Affiliated Hospital of Nanjing Medical University reviewed and permitted the use and collection of samples.

### Cell culture and transfection

MDA-MB-453, MCF-7, BT474, ZR-75-1, MDA-MB-231, HCC1806, and MCF-10A were purchased from American Type Culture Collection (ATCC), while SUM1315 was friendly and generously obtained from Stephen Ethier (University of Michigan, AnnArbor, MI, USA). MCF-10A, MDA-MB-453, MCF-7, BT474, ZR-75-1, MDA-MB-231 and SUM1315 cells lines were cultured in Dulbecco’s modified eagle medium (DMEM) (Wisent, China), and HCC1806 cell line was cultured in RPMI 1640 (Wisent, China). 10% fetal bovine serum, 100 mg/ml streptomycin and 100 U/ml penicillin were added into DMEM or RPMI 1640.

### RNA extraction and quantitative real-time polymerase chain reaction (qRT-PCR)

Total RNAs were extracted using Trizol reagent (TaKaRa, Japan). The HiScript Q RT SuperMix (Vazyme, China) was used to synthesize complementary DNAs (cDNAs). A real-time PCR instrument (Applied Biosystems, USA) was used to conduct the qRT–PCR. The abundance of targeted mRNAs and miRNAs was normalized with Glyceraldehyde-3-phosphate dehydrogenase (GAPDH) and U6. The 2^-ΔΔCT^ method or 2^-ΔCT^ method was used to calculate the relative expression. The primers were listed in Table S1.

### Lentivirus construction and cell transfection

miRNA mimics, miRNA inhibitor, mimics controls and inhibitor control were synthesized by GenePharma (Shanghai, China), as well as the small interfering RNAs (siRNAs) targeting HM13 gene. A Lipofectamine 3000 transfection reagent (Invitrogen) was added into breast cancer cells for the improvement of transfection efficiencies. Commercially available lentiviral vectors containing HM13 sequence were from Genechem (Shanghai, China). Meanwhile, mCherry-EGFP-LC3B was constructed by GenePharma (Shanghai, China). Moreover, stable pooled populations of breast cancer cells were selected using puromycin (3 μg/ml) for 2 weeks. Table S2 listed the sequence of corresponding siRNA and RNA oligonucleotides.

### Western blot analysis and main reagents

Cells and paired primary tissues were extracted by lysis buffer containing 1% phosphatase inhibitor, 1% phenylmethanesulfonyl fluoride (PMSF), and 0.1% protease inhibitor. The total proteins were separated through 10% SDS-PAGE and transferred to the PVDF membrane (Bio-Rad). And then, 2.5 g skim milk dissolved with 50 ml Tris buffered saline containing 0.1% Tween 20 (TBST) was used to block the membranes. After the incubation of the primary antibody against HM13 (1:1000, Proteintech, USA, 20416-1-AP), PERK (1:1000, Cell Signaling Technology, USA, 5683 T), CHOP (1:1000, Cell Signaling Technology, USA, 2895 T), LC3B (1:1000, Cell Signaling Technology, USA, 3868 T), p62 (1:1000, Cell Signaling Technology, USA, 16177 S), BNIP3 (1:1000, Cell Signaling Technology, USA, 44060 S), p-PI3K (1:1000, Cell Signaling Technology, USA, 17366 S), PI3K (1:1000, Cell Signaling Technology, USA, 4249 T), p-AKT (1:1000, Cell Signaling Technology, USA, 4060 T), AKT (1:1000, Cell Signaling Technology, USA, 4691 T), p-mTOR (1:1000, Cell Signaling Technology, USA, 5536 T), mTOR (1:1000, Cell Signaling Technology, USA, 2983 T), GAPDH (1:1000, Beyotime, China, AF1186) and secondary antibodies, the relative protein expression levels derived from the bands were detected by an ECL detection system. GAPDH was used for the normalization of protein loading. The antibodies were all diluted according to product usage information. All tests of western blots were performed in triplicate. Chloroquine (CQ, HY-17589A) and insulin-like growth factor I (IGF-1, HY-P7018) were purchased from MedChemExpress, Monmouth, Junction, NY.

### CCK-8 assay

Cell proliferation was assessed by using CCK-8 kit (Vazyme, China), following the manufacturer’s instruction. In brief, 2000 targeted breast cancer cells were seeded into a 96-well plate and cultured with DMEM containing 10% FBS for 5 days. At the certain time of the day, 100 μl fresh medium (90 μl DMEM and 10 μl CCK-8) was added into 96-well plates and the target cells were incubated for 2.5 h at 37 °C. The absorbance was finally detected at 450 nm with a microplate reader (Groding, Tecan, Austria). Each test was performed in triplicate.

### Colony formation assay

A total of 500 cells were plated into 6-well plates and cultured for 2 weeks. The colonies were then fixed with paraform and stained with 1% crystal violet (Beyotime, China). After drying them at room temperature, we counted the numbers of colonies by using Image J counting particle tools and indicated the results as a bars graph.

### EdU assay

EdU assay was conducted to evaluate the proliferation ability of breast cancer cells. After seeded into 96-well plates (2 × 10^4^ cells/well) with DMEM for 24 h, breast cancer cells were treated with 50 μM EdU at 37 °C for 2 h. After the treatment of 4% paraformaldehyde and 0.5% Triton X-100, those targeted cells were stained with 1× Apollo® reaction cocktail for 30 min. Ultimately, nuclei got stained by 1 × 2-(4-amidinophenyl)-6-indolecarbamidine dihydrochloride (DAPI). The cells were visualized under a fluorescence microscope (Nikon, Japan).

### Tumor subcutaneous mice model

All animal experiments involved in this study were permitted by Institutional Animal Care and Use Committee of the Nanjing Medical University. 1 × 10^7^ SUM1315 cells were subcutaneously injected into nude mice (aged 4 weeks, female), which were randomly divided into four groups (sh-NC, sh-HM13, Vector and HM13). Tumor volume [Volume = (length × width^2^)/2] was measured once a week. Lastly, the mice were euthanised after 4 weeks. Meanwhile, their final tumor weight was checked.

### Immunohistochemistry (IHC) staining and analysis

The samples from tumor subcutaneous mice model were blocked with 10% formalin and then embedded in paraffin. The sections were treated with specific primary antibodies. The antibody of HM13 (Proteintech, USA) and Ki-67 (Proteintech, USA) were used in the process of IHC staining. The nuclei were counterstained with hematoxylin for the final step, after which we photographed the slides through a microscope.

### Fluorescent confocal microscopy

SUM1315 and ZR-75-1 cell lines were stably transfected by tandem mCherry-EGFP-LC3B and further infected with siRNAs targeting HM13 gene, as well as their corresponding blank control. After that, cells were seeded at a density of 5 × 10^3^ cells for 24 h incubation. The cells were then immobilized by 4% paraformaldehyde with 0.5% Triton X-100. After all of this, the nucleuses were stained with DAPI. Finally, they were observed at indicated time under the confocal microscope.

### Transmission electron microscopy (TEM)

Breast cancer cells (2 × 10^5^ per well) were seeded in 6-well plates and cultured for 16 h. After transfection of siRNAs targeting HM13 gene, breast cancer cells were collected and immobilized with a solution containing 2% paraformaldehyde, following 1% osmium tetroxide for 1 h. The samples were dehydrated with a graded alcohol series, and negatively stained with phosphotungstic acid. Finally, the ultrastructure of the prepared cells was observed by electron microscope (JEOL JEM-1400Flash).

### Transwell assay

Breast cancer cell abilities of migration and invasion were conducted by using 24-well transwell inserts (Millicell Hanging Cell Culture Insert, USA) coated without or with Matrigel (BD Biosciences, USA). Up of the membrane pore of the transwell chamber was seeded by 200 μl cell suspension with serum-free DMEM. And then, 600 μl medium with 10% FBS was a chemoattractant for cells from the upper side. Cells were incubated at 37 °C for 24 h or 48 h, after which the successfully invading or migrating cells were finally stained with 1% crystal violet for analysis.

### Cell wound-healing assay

Cells were cultured in 6-well plates until the subfusion state. Afterwards, 200 μl sterile pipette tip was used as a tool to create the linear scratch. Cell debris were then washed by phosphate-buffered saline (PBS, Hyclone, USA) and images were captured at 0 h and 48 h by inversion fluorescence microscopy (Olympus, Japan). Migration area [Migration area% = area of (0 h–48 h)/area of 0 h]. All tests were performed in triplicate.

### Dual-luciferase reporter assay

Predicted binding sites between HM13 and miR-760 were synthesized and cloned inside a dual luciferase reporter vector by pmirGLO luciferase vector (Promega, USA). The cells in 96-well plates were co-transfected with either miR-760 mimics or mimics control with the reporter gene. And then, we measured the relative luciferase activity according to the manufacture’s procedure (Promega, USA).

### Statistical analysis

All the experiments were performed in triplicate, unless otherwise specified. Moreover, the data were analyzed with Pearson χ^2^ tests, Student’s *t*-test and ANOVA using SPSS statistical software. A value of *P* < 0.05 was considered statistically significant.

## Supplementary information

Checklist-CDDIS-22-0296RR

DECLARATION OF CONTRIBUTIONS TO ARTICLE

Supplementary Material

Figure S1

Figure S2

Figure S3

Figure S4

Figure S5

Figure S6

Original western blots

Table S2

## Data Availability

The datasets used and/or analyzed during the current study are available from the corresponding author on reasonable request.
