# Supplementary material for: Histocompatibility Minor 13 (HM13), targeted by miR-760, exerts oncogenic role in breast cancer by suppressing autophagy and activating PI3K-AKT-mTOR pathway
Source: Cell Death Dis. 2022 Sep 25;13(8):728. doi: 10.1038/s41419-022-05154-4 (PMC9509374; doi:10.1038/s41419-022-05154-4)
Supplement: Supplementary file 3 — Supplementary Material [file 41419_2022_5154_MOESM3_ESM.docx]

**Figure legends**

**Figure S1.** **HM13 is related to poor prognosis of breast cancer patients**. **A**-**B** Higher expression of HM13 was associated with poorer PPS (A) and DMFS (B) of breast cancer patients based on TCGA database.

**Figure S2. Upregulated HM13 facilitates the proliferation of breast cancer *in vitro* and *in vivo*. A-B** qRT-PCR (A) and western blot (B) were used to estimate the transfection efficiencies of HM13 overexpression in breast cancer cells, respectively. **C-F** Cell proliferation abilities in breast cancer were evaluated by CCK8 (C), colony formation (D) and EdU assays (E-F). Scale bars, 50 µm. **G** Tumor tissues of HM13 overexpression (HM13) and control (Vector) groups (n = 6) were excised and photographed. **H-I** Tumor growth (H) and tumor weight (I) of nude mice subcutaneously injected by stably transfected SUM1315 cells were monitored and presented by tumor growth curves. **J** Representative photos of IHC staining harvested from tumor tissues of nude mice were presented to characterize the positive rates of HM13 and Ki-67. Scale bars, 100 µm. Data were shown as mean ± SD, *p < 0.05, **p < 0.01.

**Figure S3. HM13 enhances the breast cancer metastasis abilities in vitro. A-B** siRNAs (si-HM13-1 and si-HM13-2) or negative control (si-NC) were transfected into SUM1315 (A) and ZR-75-1 (B) cell lines. The wound healing assays were performed to evaluate the migration abilities in breast cancer. Scale bars, 100 µm. **C-D** The metastasis abilities of SUM1315 and ZR-75-1 cell lines were detected via transwell assays. Scale bars, 50 µm. **E-F** HM13 overexpression (HM13) or control (Vector) was stably transfected into SUM1315 (E) and ZR-75-1 (F) cell lines, respectively. The cell migration abilities were assessed by the wound healing assays at 0 h and 48 h. Scale bars, 100 µm. **G-H** The breast cancer cell migration (G) and invasion (H) abilities in HM13 overexpression (HM13) and control (Vector) group were examined by transwell assay. Scale bars, 50 µm. Data were shown as mean ± SD, *p < 0.05, **p < 0.01.

**Figure S4. miR-760 inhibits breast cancer cell proliferation. A** The mRNA expression of miR-760 was downregulated in breast cancer tissues compared to adjacent normal tissues. **B** Negative correlation between the mRNA expression of HM13 and miR-760 in breast cancer tissues. **C-F** Cell viability of SUM1315 and ZR-75-1 was estimated by CCK-8 assay (C-D) and EdU assay (E-F). SUM1315 and ZR-75-1 cell lines were transfected with mimics control (miR-NC), miR-760 mimics (miR-760), inhibitor control (inh-NC) or miR-760 inhibitor (inh-760). Scale bars, 50 µm. Data were shown as mean ± SD, *p < 0.05, **p < 0.01, ***p < 0.001.

**Figure S5. miR-760 suppresses breast cancer cell metastasis. A-B** Representative images of the wound healing assays using SUM1315 (A) and ZR-75-1 (B) transfected with mimics control (miR-NC), miR-760 mimics (miR-760), inhibitor control (inh-NC) or miR-760 inhibitor (inh-760). Scale bars, 100 µm. **C-D** The migration (C) and invasion (D) abilities of the same set of cell lines transfected with mimics control (miR-NC), miR-760 mimics (miR-760), inhibitor control (inh-NC) or miR-760 inhibitor (inh-760) were analysed by transwell assays. Scale bars, 50 µm. Data were shown as mean ± SD, *p < 0.05.

**Figure S6. FKBP8 was degraded by HM13.** Western blot was used to estimate the protein level of HM13 and FKBP8 in SUM1315 and ZR-75-1, respectively. The western blots were performed in triplicate.

**Table S1: The primer sequence of qRT-PCR**

| Name | Sense（5’-3’） |
| --- | --- |
| HM13 | F:5’-AGCTCCTGCACCTCAACAAT-3’  R:5’-ACATCTCCAAGTCCCAGCAT-3’ |
| BIP | F:5’-GCTATTGCTTATGGCCTGGA-3’  R:5’-CTGACATCTTTGCCCGTCTT-3’ |
| CHOP | F:5’-AACCAGGAAACGGAAACAGA-3’  R:5’-TCACCATTCGTCAATCAGA-3’ |
| IRE1-A | F:5’-GTCAACGCTGGATGGAAGTT-3’  R:5’-GATGCCTGCACCAATTCTG-3’ |
| ATF6 | F:5’-CAGCGAATAGCCCAGTGAAT-3’  R:5’-TCTCGCCTCTAACCCTAGCA-3’ |
| PERK | F:5’-GACCAAGACCGTGAAAGCAT-3’  R:5’-CGCTGTAGAAGCAGGATGTC-3’ |
| miR-760 | F:5’-TATTGCTTAAGAATACGCGTAG-3’  R:5’-AACTCCAGCAGGACCATGTGAT-3’ |
| GAPDH | F:5’- GAAGGTGAAGGTCGGAGTC-3’  R:5’- GAAGATGGTGATGGGATTTC-3’ |
| U6 | F: 5’-CTCGCTTCGGCAGCACA-3’  R: 5’-AACGCTTCACGAATTTGCGT-3’ |

**Table S2: siRNA and RNA oligonucleotides sequences**

| Name | Sense（5’-3’） |
| --- | --- |
| si-HM13-1 | UGCCUGAAACAAUCACCAGC |
| si-HM13-2 | GCAGGAGGUUGAUGUACUCC |
| miR-760 mimics | CGGCUCUGGGUCUGUGGGGA |
| miR-149-5p mimics | UCUGGCUCCGUGUCUUCACUCCC |
| miR-6822-3p mimics | AGGCUCUAACUGGCUUUCCCUGCA |
| miR-6747-3p mimics | UCCUGCCUUCCUCUGCACCAG |
| mimics control | UUCUCCGAACGUGUCACGUTT |
| miR-760 inhibitor | UCCCCACAGACCCAGAGCCG |
| inhibitor control | CAGUACUUUUGUGUAGUACAA |
