# Supplementary figures and images for: Histocompatibility Minor 13 (HM13), targeted by miR-760, exerts oncogenic role in breast cancer by suppressing autophagy and activating PI3K-AKT-mTOR pathway
Source: Cell Death Dis. 2022 Sep 25;13(8):728. doi: 10.1038/s41419-022-05154-4 (PMC9509374; doi:10.1038/s41419-022-05154-4)

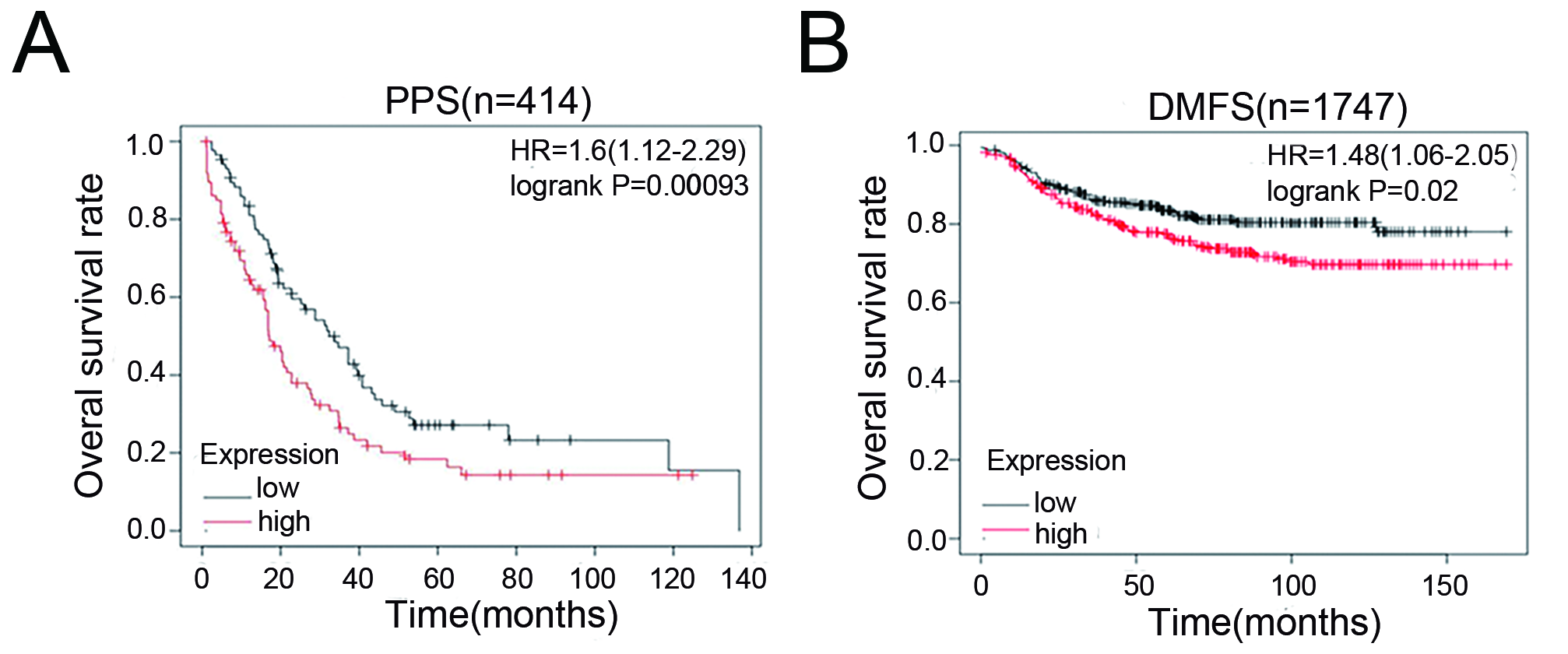

Supplement: Supplementary file 4 — Figure S1 [file 41419_2022_5154_MOESM4_ESM.tif]

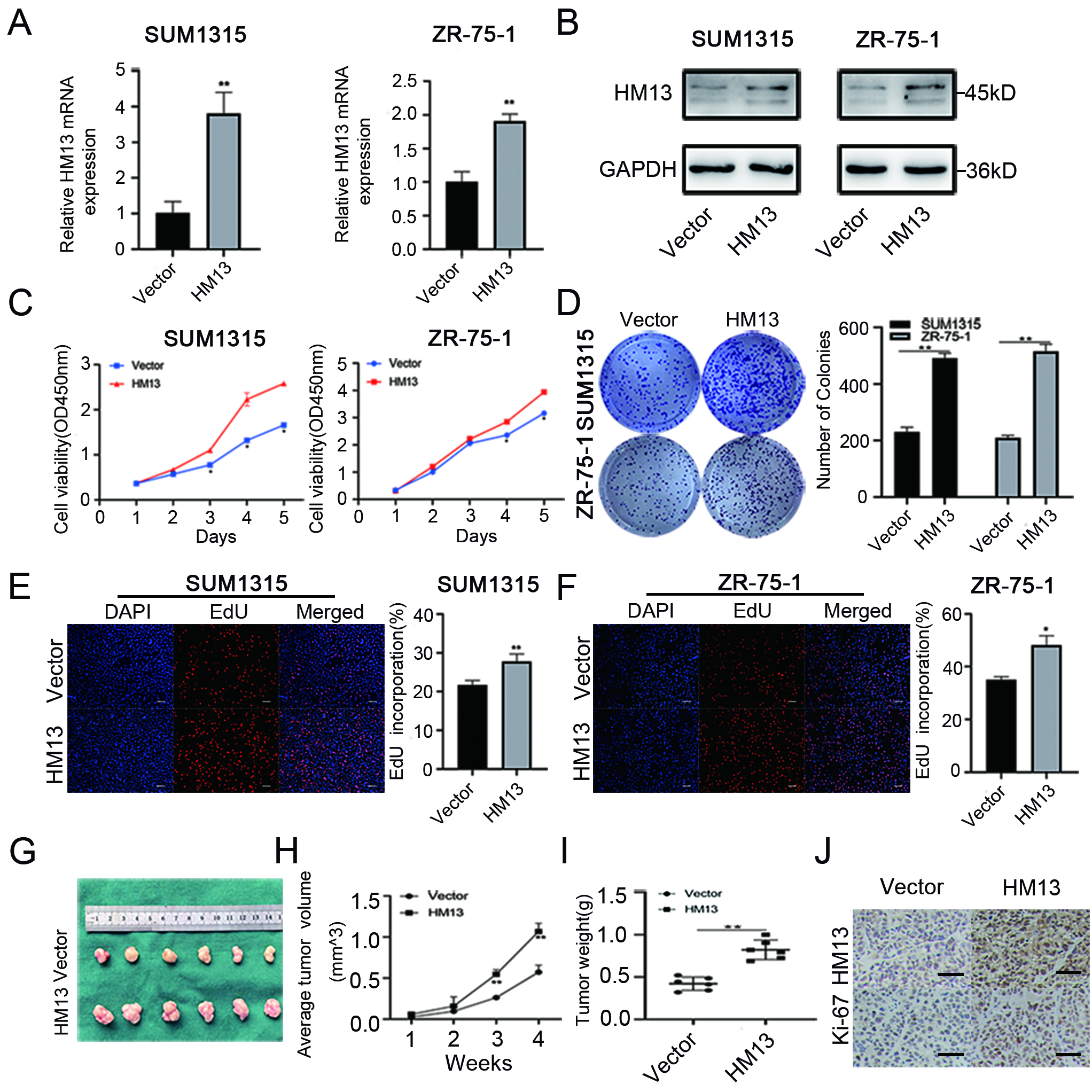

Supplement: Supplementary file 5 — Figure S2 [file 41419_2022_5154_MOESM5_ESM.tif]

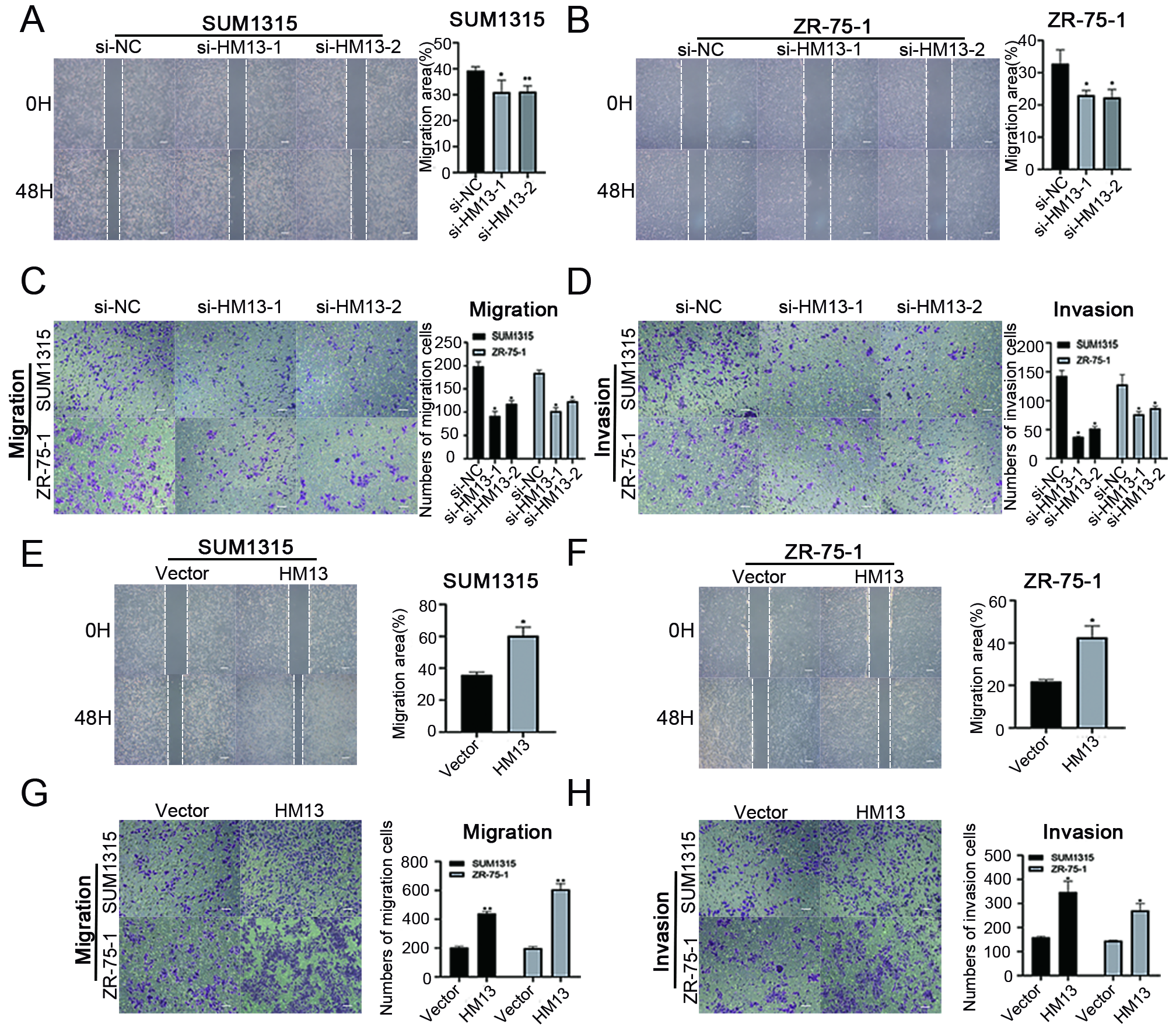

Supplement: Supplementary file 6 — Figure S3 [file 41419_2022_5154_MOESM6_ESM.tif]

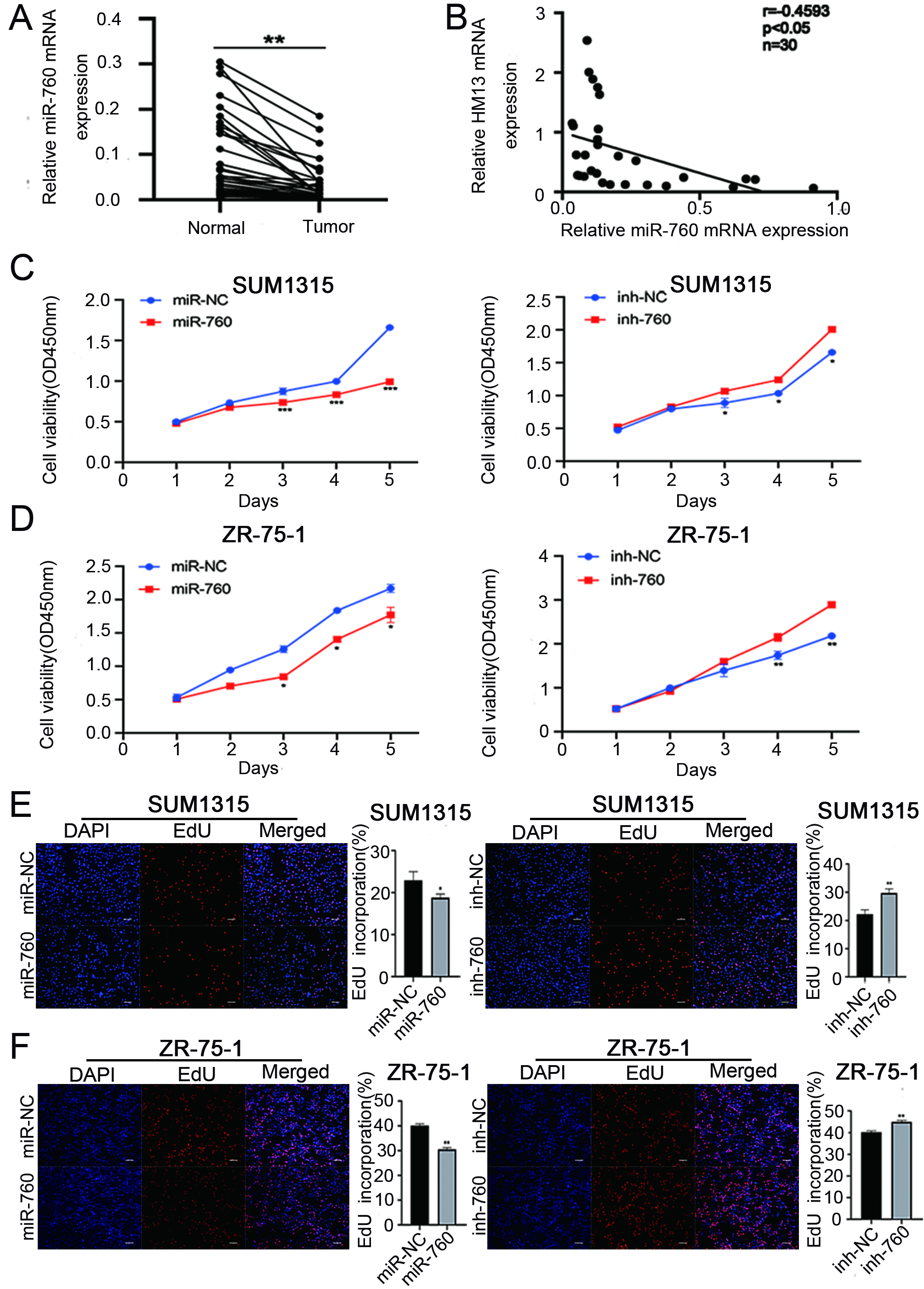

Supplement: Supplementary file 7 — Figure S4 [file 41419_2022_5154_MOESM7_ESM.tif]

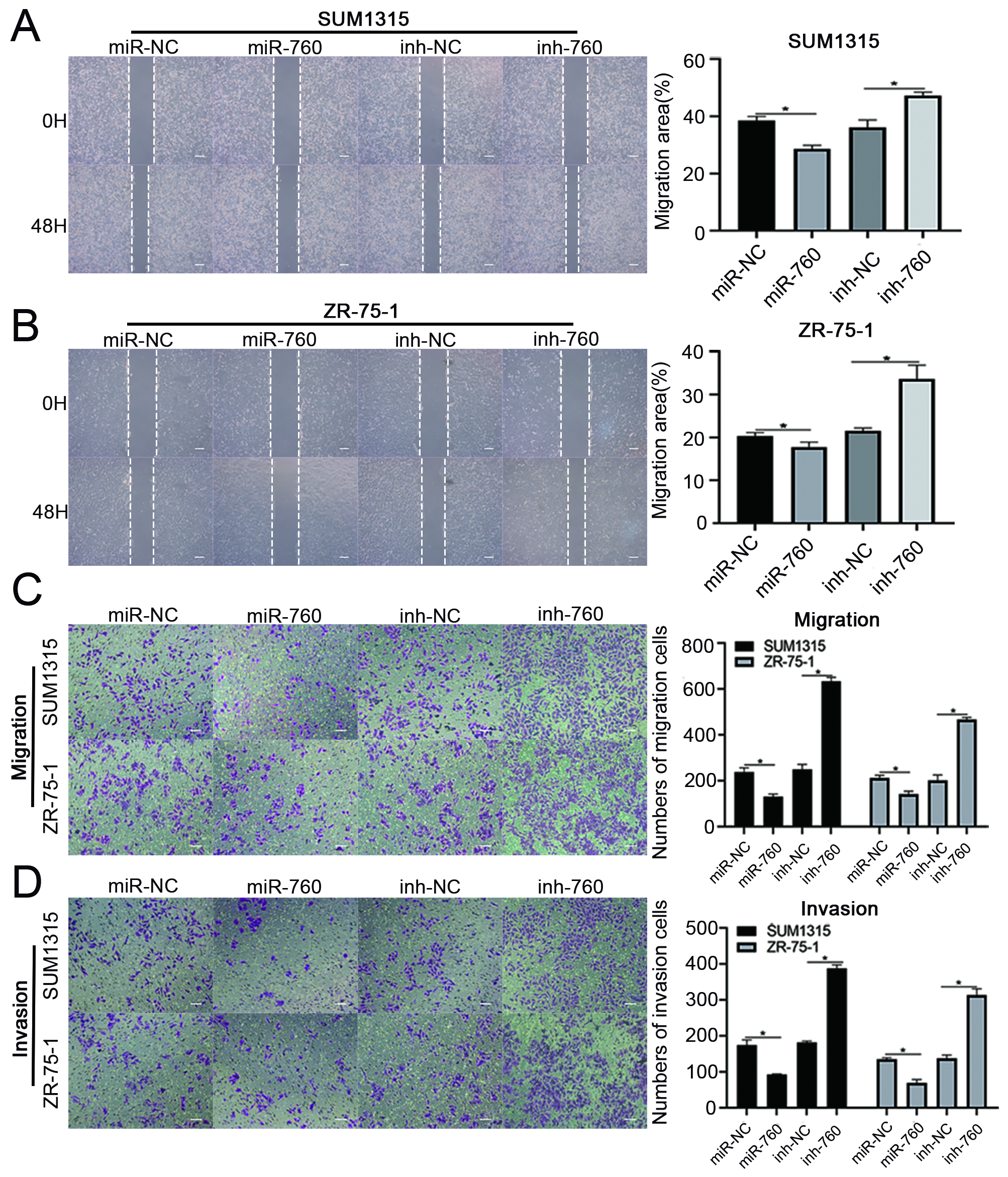

Supplement: Supplementary file 8 — Figure S5 [file 41419_2022_5154_MOESM8_ESM.tif]

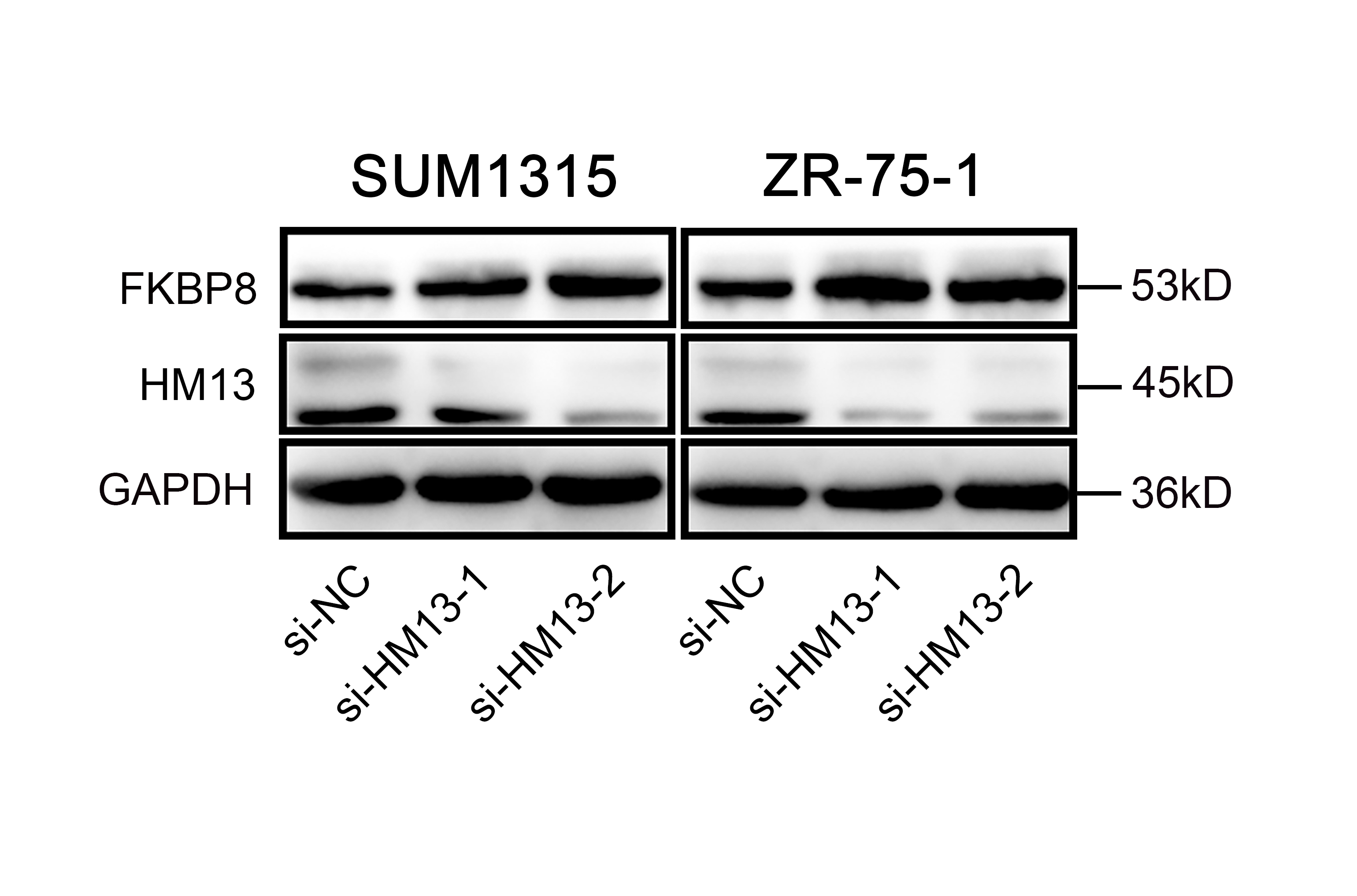

Supplement: Supplementary file 9 — Figure S6 [file 41419_2022_5154_MOESM9_ESM.tif]
