# Supplementary material for: Histocompatibility Minor 13 (HM13), targeted by miR-760, exerts oncogenic role in breast cancer by suppressing autophagy and activating PI3K-AKT-mTOR pathway
Source: Cell Death Dis. 2022 Sep 25;13(8):728. doi: 10.1038/s41419-022-05154-4 (PMC9509374; doi:10.1038/s41419-022-05154-4)
Supplement: Supplementary file 10 — Original western blots [file 41419_2022_5154_MOESM10_ESM.pdf]

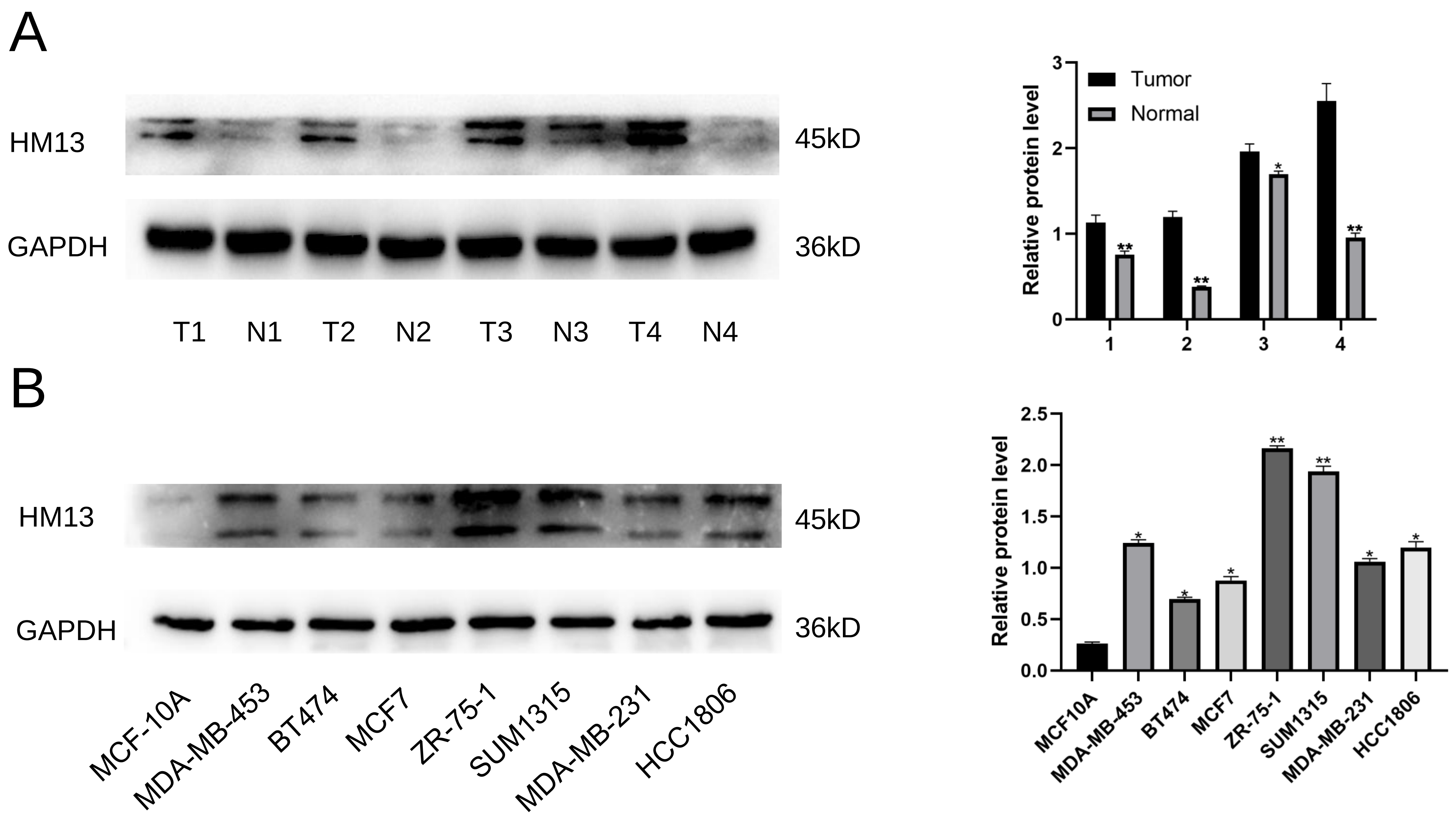
A
HM13
GAPDH
45kD
36kD
T1 N1 T2 N2 T3 N3 T4 N4
Tumor
Normal
Relative protein level
0
1
2
3
1 2 3 4
*
**
B
HM13
GAPDH
45kD
36kD
MCF-10A
MDA-MB-453
BT474
MCF7
ZR-75-1
SUM1315
MDA-MB-231
HCC1806
Relative protein level
0.0
0.5
1.0
1.5
2.0
2.5
MCF10A
MDA-MB-453
BT474
MCF7
ZR-75-1
SUM1315
MDA-MB-231
HCC1806

C

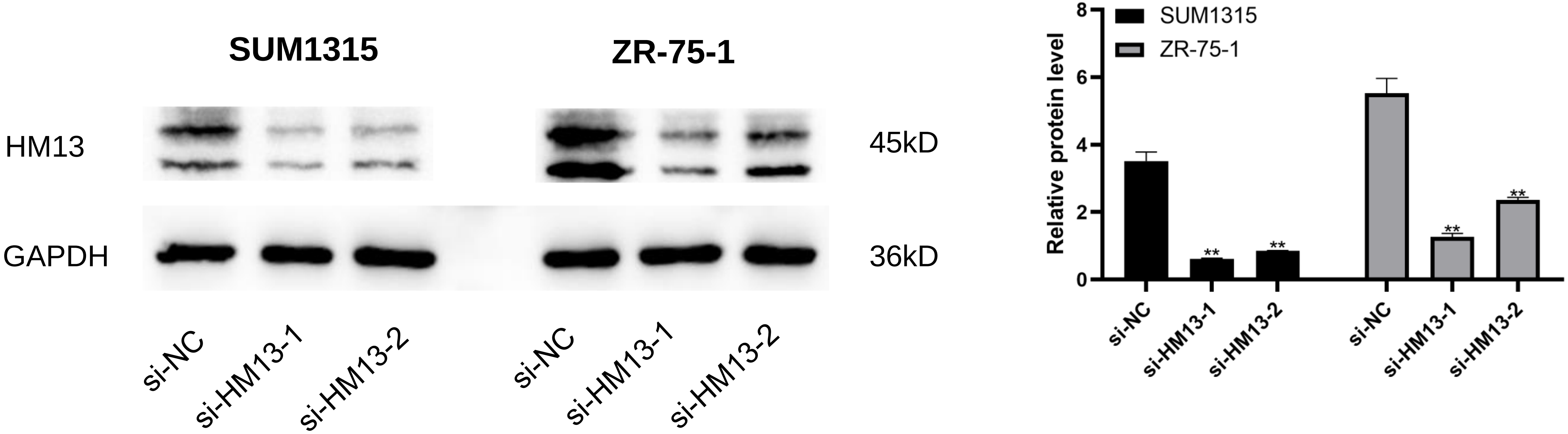
SUM1315
ZR-75-1
HM13
GAPDH
45kD
36kD
si-NC
si-HM13-1
si-HM13-2
si-NC
si-HM13-1
si-HM13-2
SUM1315
ZR-75-1
Relative protein level
0
2
4
6
8
**
**
**
**
si-NC
si-HM13-1
si-HM13-2
si-NC
si-HM13-1
si-HM13-2

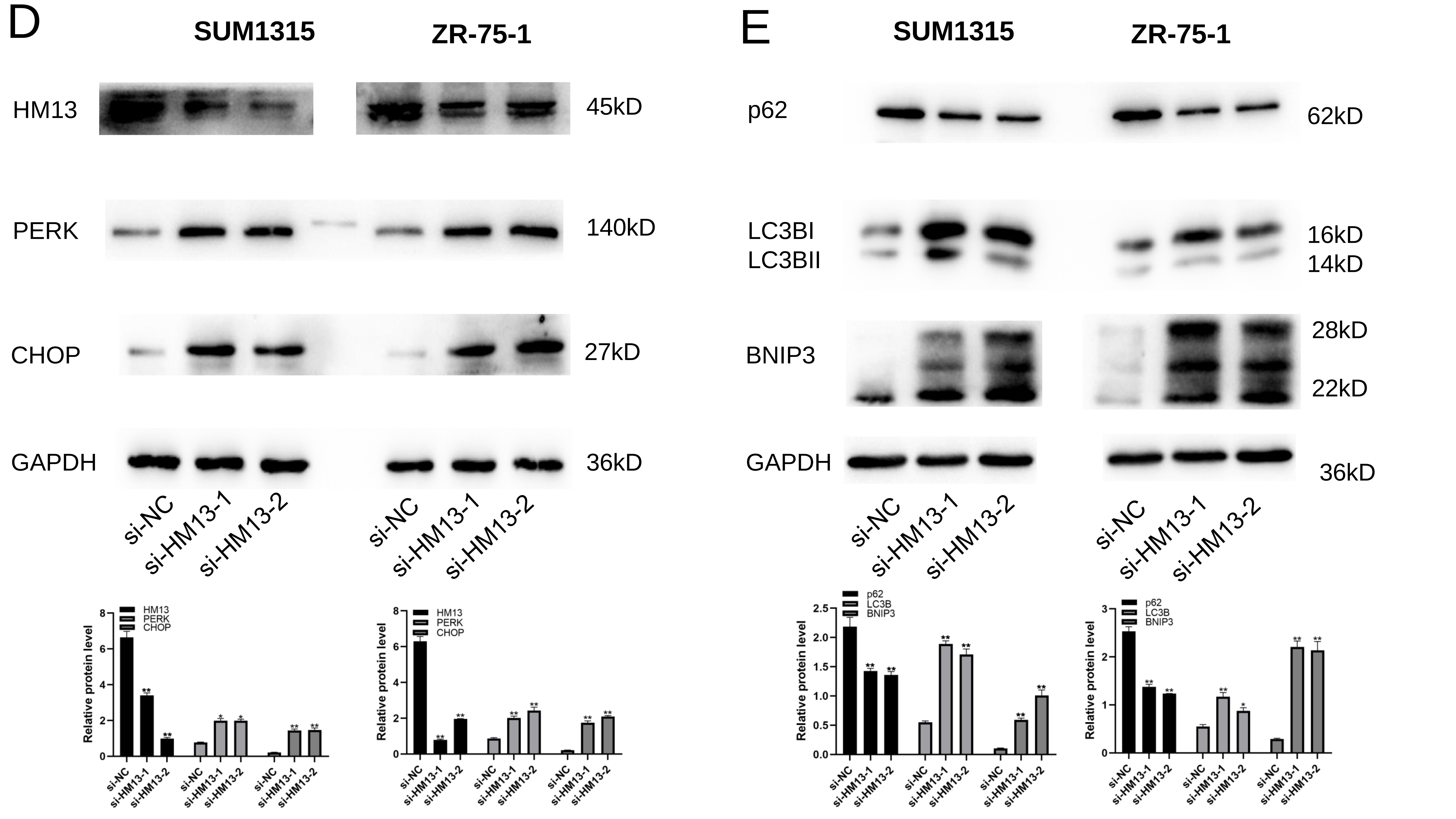

D
SUM1315
ZR-75-1
HM13
45kD
PERK
140kD
CHOP
27kD
GAPDH
36kD
si-NC
si-HM13-1
si-HM13-2
HM13
PERK
CHOP
Relative protein level
E
SUM1315
ZR-75-1
p62
62kD
LC3BI
LC3BII
16kD
14kD
BNIP3
28kD
22kD
GAPDH
36kD
si-NC
si-HM13-1
si-HM13-2
p62
LC3B
BNIP3
Relative protein level

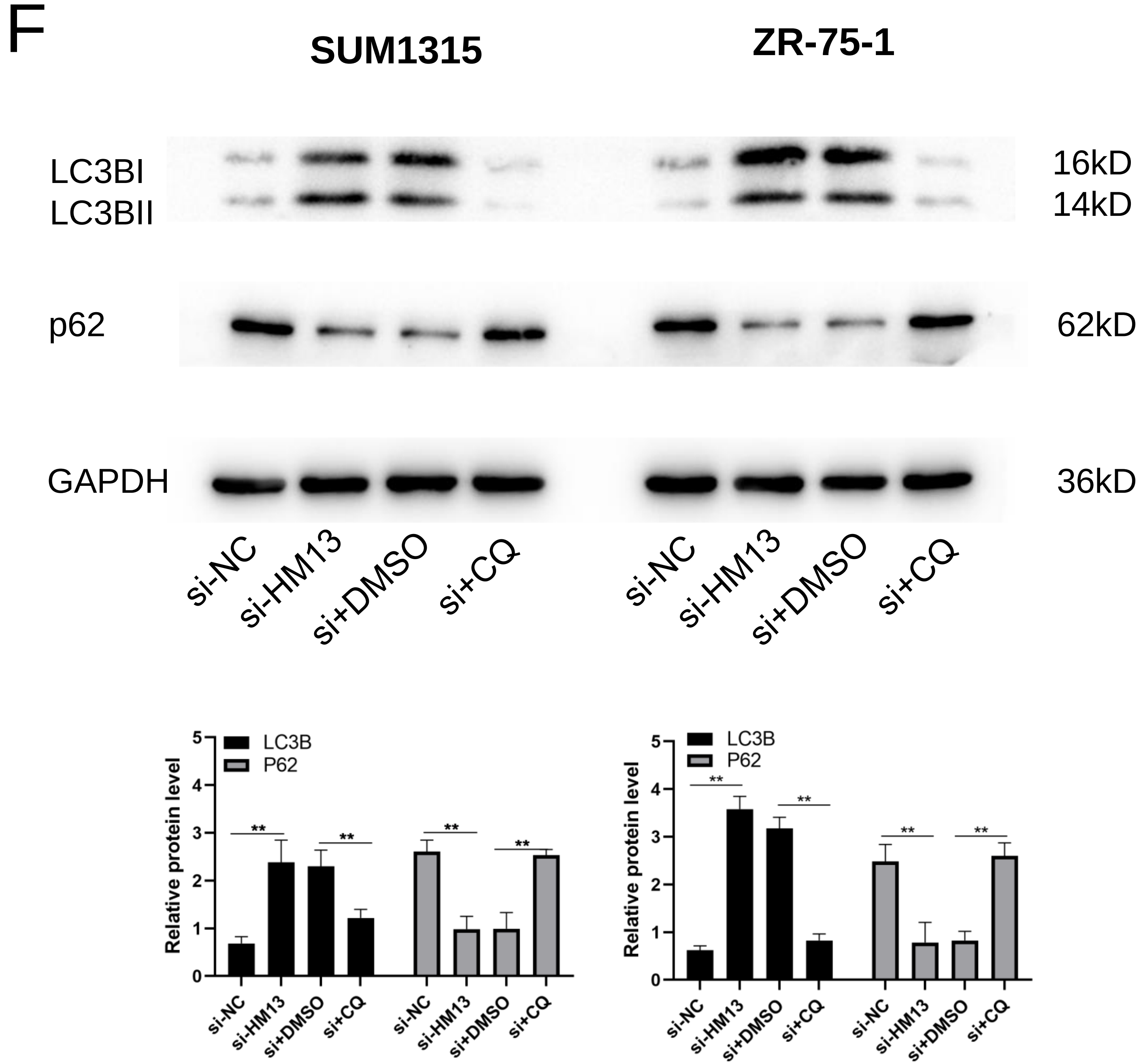
F
SUM1315
ZR-75-1
LC3BI
LC3BII
16kD
14kD
p62
62kD
GAPDH
36kD
si-NC
si-HM13
si+DMSO
si+CQ
LC3B
P62
Relative protein level
**

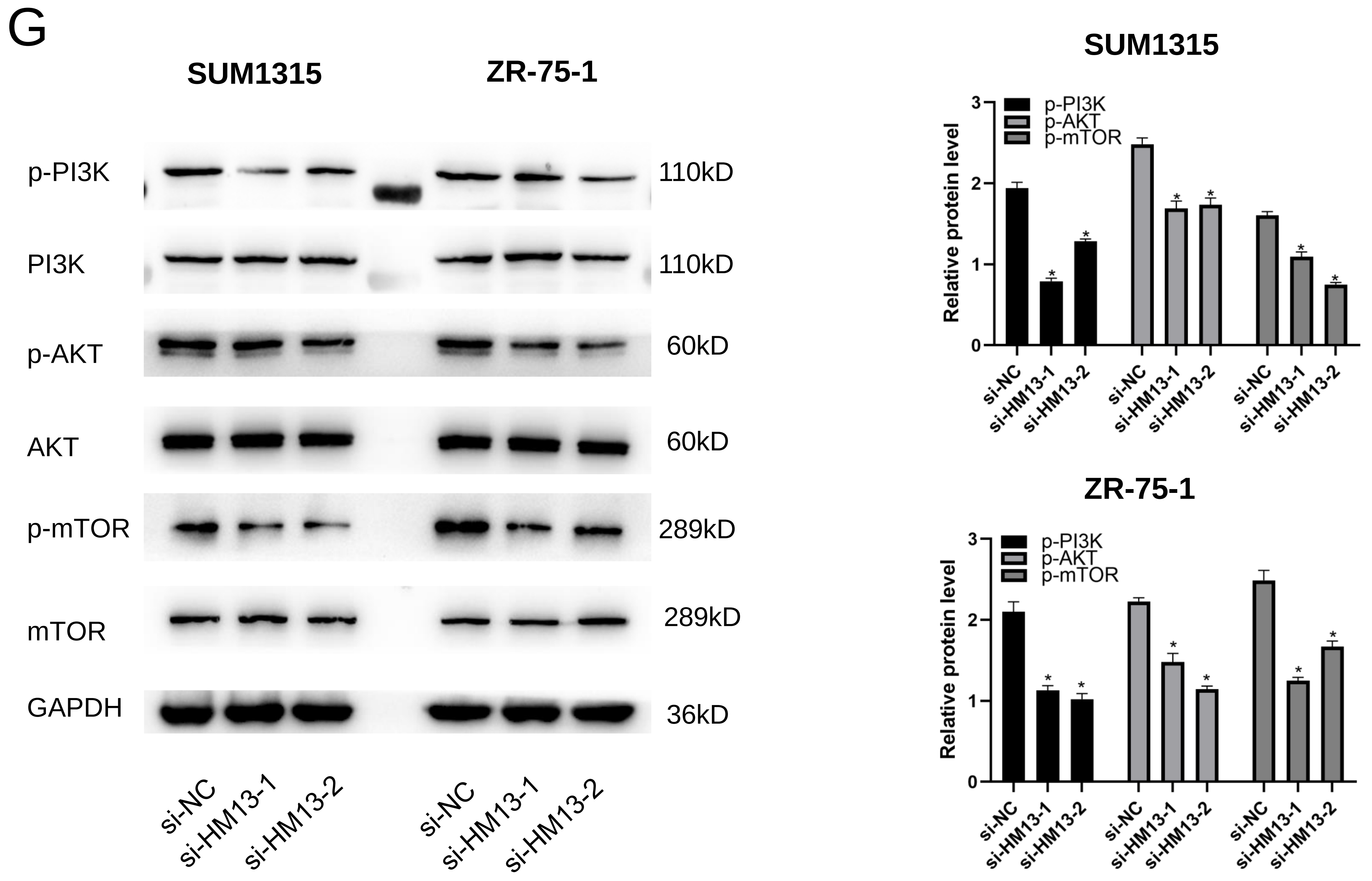
G
SUM1315
ZR-75-1
p-PI3K
PI3K
p-AKT
AKT
p-mTOR
mTOR
GAPDH
110kD
110kD
60kD
60kD
289kD
289kD
36kD
si-NC
si-HM13-1
si-HM13-2
si-NC
si-HM13-1
si-HM13-2
SUM1315
Relative protein level
p-PI3K
p-AKT
p-mTOR
ZR-75-1
Relative protein level
p-PI3K
p-AKT
p-mTOR

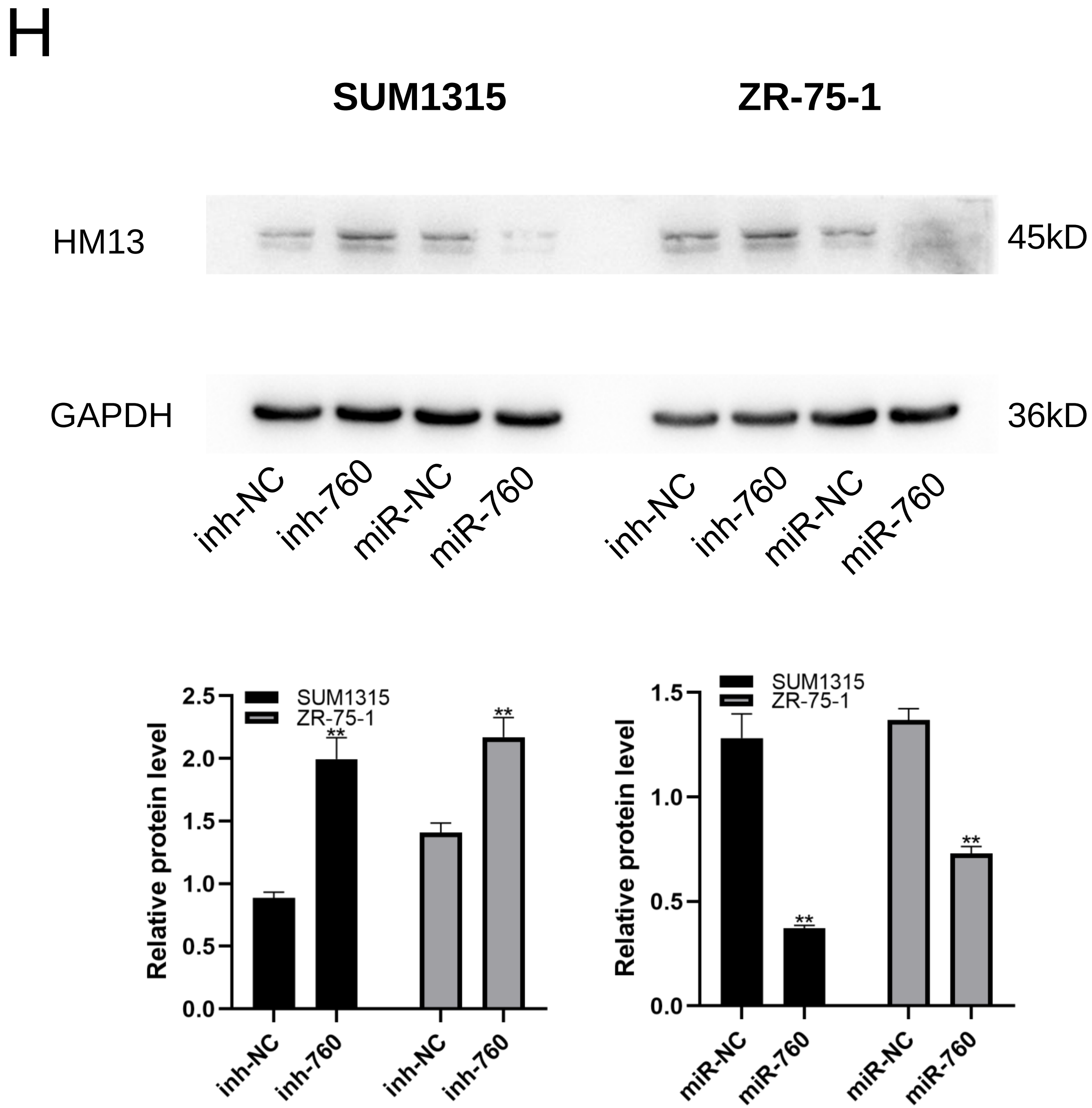

H
SUM1315
ZR-75-1
HM13
45kD
GAPDH
36kD
inh-NC
inh-760
miR-NC
miR-760
inh-NC
inh-760
miR-NC
miR-760
SUM1315
ZR-75-1
Relative protein level
2.5
2.0
1.5
1.0
0.5
0.0
**
**
inh-NC
inh-760
inh-NC
inh-760
SUM1315
ZR-75-1
Relative protein level
1.5
1.0
0.5
0.0
**
**
miR-NC
miR-760
miR-NC
miR-760

I

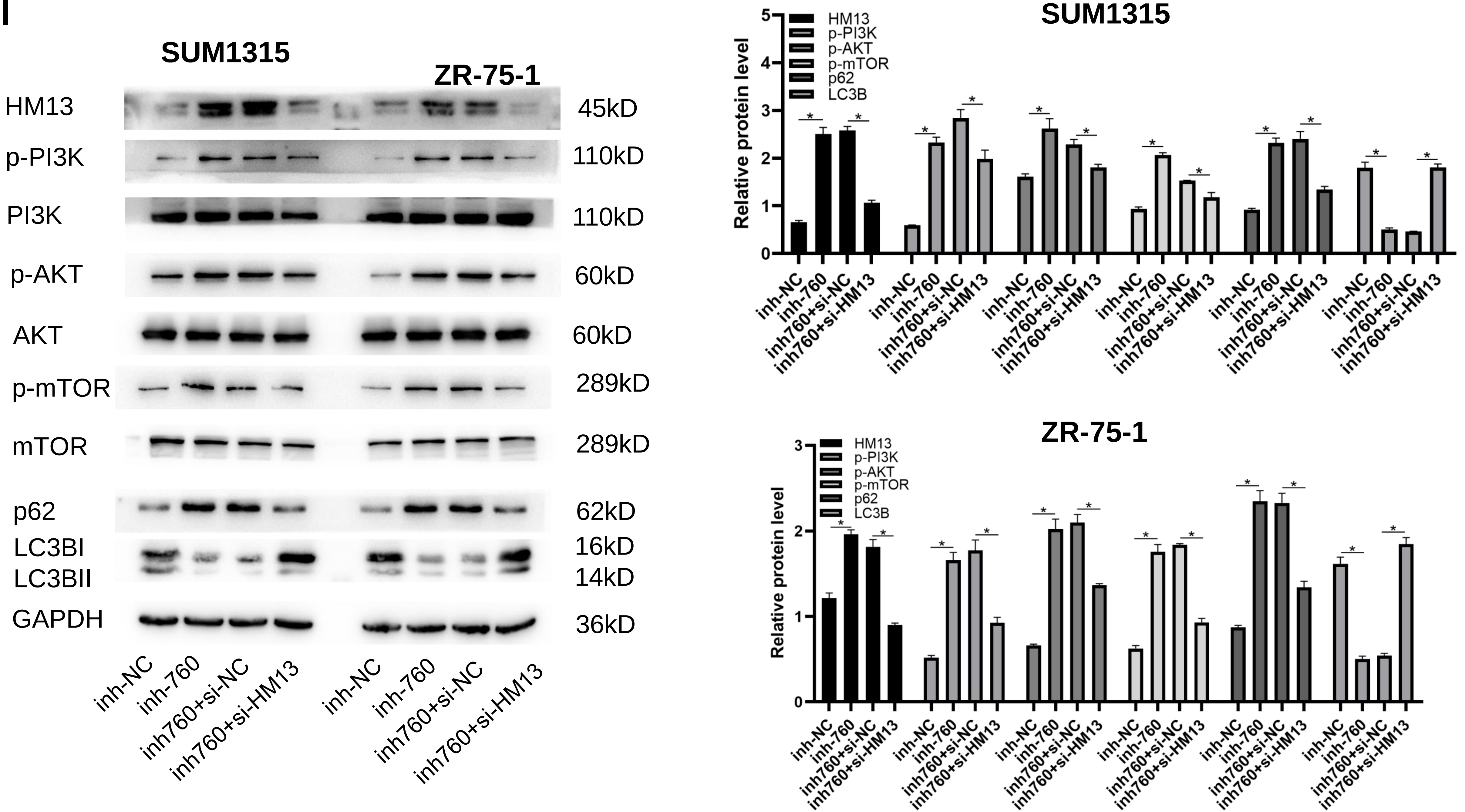
SUM1315
ZR-75-1
HM13
p-PI3K
PI3K
p-AKT
AKT
p-mTOR
mTOR
p62
LC3BI
LC3BII
GAPDH
45kD
110kD
110kD
60kD
60kD
289kD
289kD
62kD
16kD
14kD
36kD
inh-NC
inh-760
inh760+si-NC
inh760+si-HM13
inh-NC
inh-760
inh760+si-NC
inh760+si-HM13
SUM1315
HM13
p-PI3K
p-AKT
p-mTOR
p62
LC3B
Relative protein level
ZR-75-1
HM13
p-PI3K
p-AKT
p-mTOR
p62
LC3B
Relative protein level

J

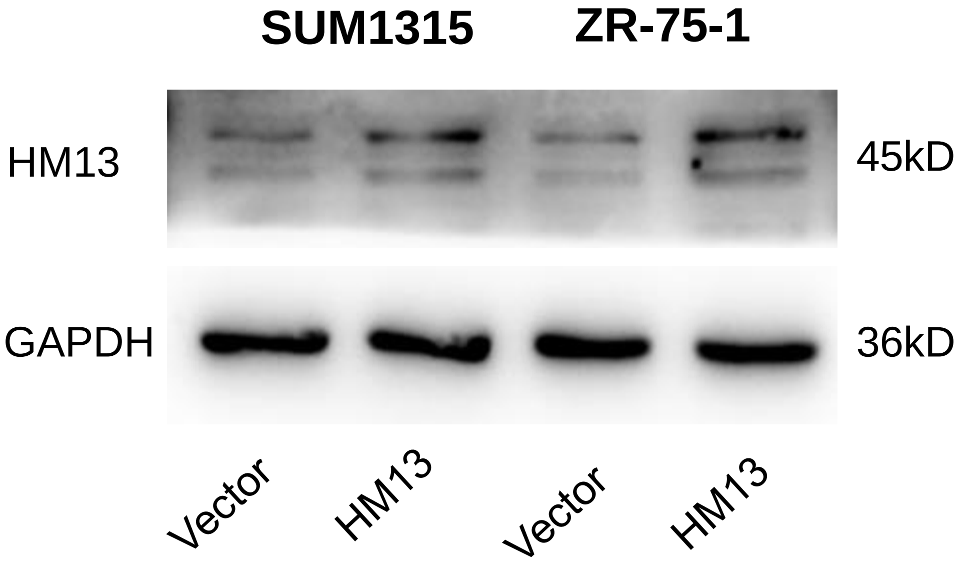
SUM1315
ZR-75-1
HM13
45kD
GAPDH
36kD
Vector
HM13
Vector
HM13

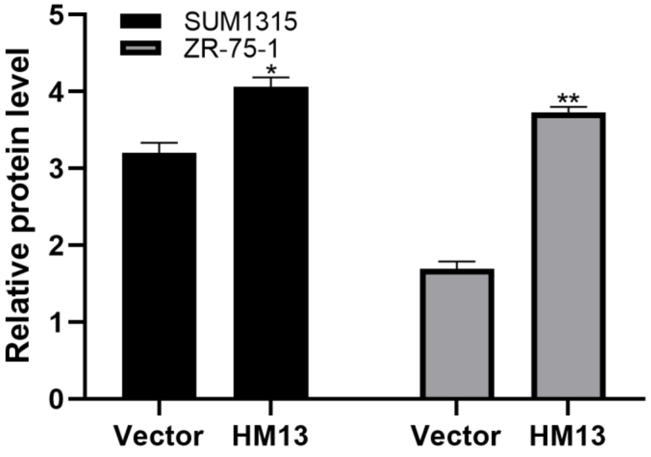
SUM1315
ZR-75-1
Relative protein level
5
4
3
2
1
0
*
**
Vector
HM13
Vector
HM13

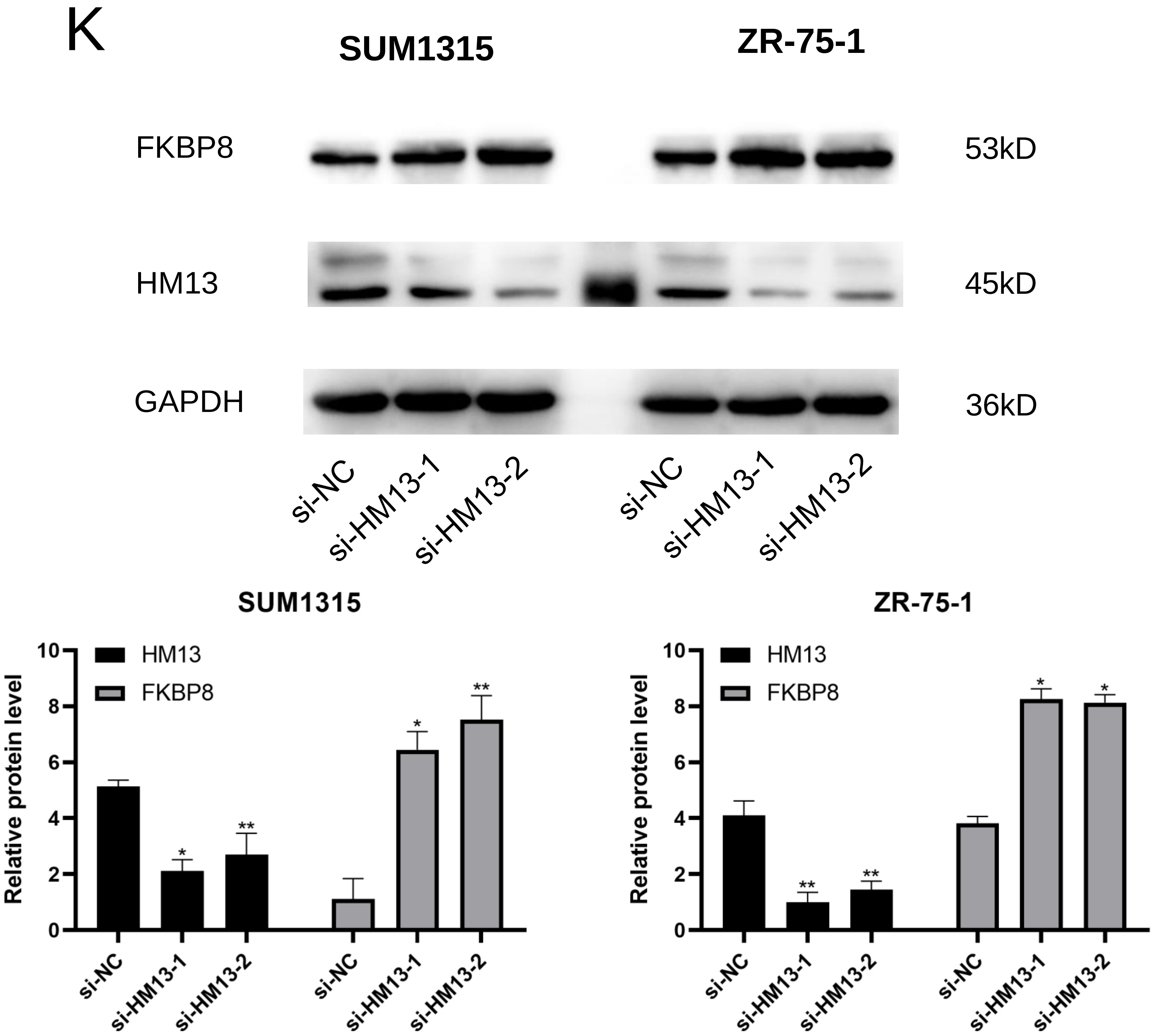
K
SUM1315
ZR-75-1
FKBP8
HM13
GAPDH
53kD
45kD
36kD
si-NC
si-HM13-1
si-HM13-2
si-NC
si-HM13-1
si-HM13-2
SUM1315
ZR-75-1
Relative protein level
HM13
FKBP8
0
2
4
6
8
10
si-NC
si-HM13-1
si-HM13-2
si-NC
si-HM13-1
si-HM13-2
